# Accelerated Lignocellulosic Molecule Adsorption Structure Determination

**DOI:** 10.1021/acs.jctc.3c01292

**Published:** 2024-02-26

**Authors:** Joakim S. Jestilä, Nian Wu, Fabio Priante, Adam S. Foster

**Affiliations:** † Department of Applied Physics, Aalto University, 00076 Aalto, Espoo, Finland; ‡ Nano Life Science Institute (WPI-NanoLSI), Kanazawa University, 920-1192 Kanazawa, Japan

## Abstract

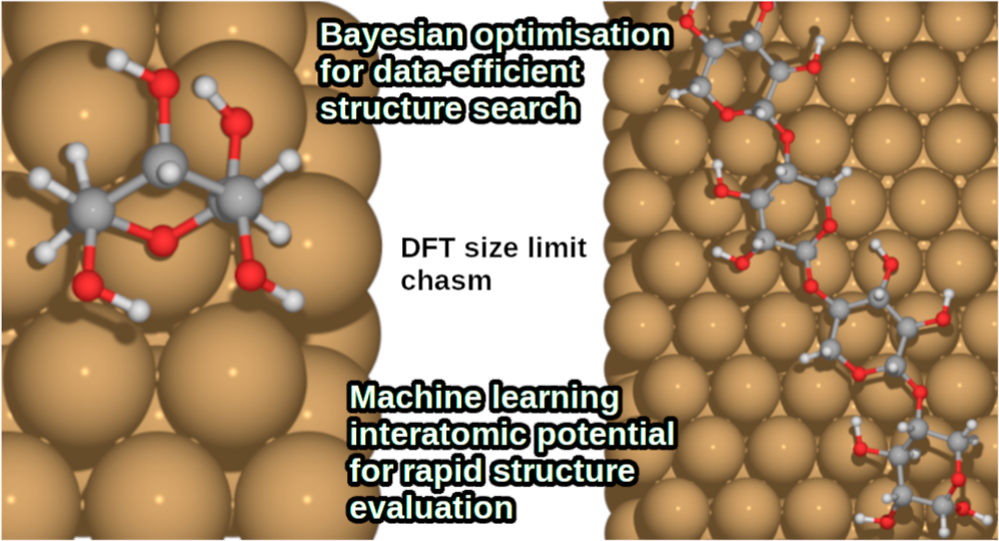

Here, we present a study combining Bayesian optimization structural inference with the machine learning interatomic potential Neural Equivariant Interatomic Potential (NequIP) to accelerate and enable the study of the adsorption of the conformationally flexible lignocellulosic molecules β-d-xylose and 1,4-β-d-xylotetraose on a copper surface. The number of structure evaluations needed to map out the relevant potential energy surfaces are reduced by Bayesian optimization, while NequIP minimizes the time spent on each evaluation, ultimately resulting in cost-efficient and reliable sampling of large systems and configurational spaces. Although the applicability of Bayesian optimization for the conformational analysis of the more flexible xylotetraose molecule is restricted by the sample complexity bottleneck, the latter can be effectively bypassed with external conformer search tools, such as the Conformer-Rotamer Ensemble Sampling Tool, facilitating the subsequent lower-dimensional global minimum adsorption structure determination. Finally, we demonstrate the applicability of the described approach to find adsorption structures practically equivalent to the density functional theory counterparts at a fraction of the computational cost.

## 1 Introduction

Determination of the global minimum of a molecular or atomistic system remains an active area of research, even with well-established methods such as basin hopping,^1^ minima hopping,^2^ and simulated annealing.^3^ Semiempirical methods are often employed in conjuncture with these algorithms, but these are not necessarily sufficiently accurate—or even available—for the system of interest. Being more accurate than semiempirical methods, density functional theory (DFT) is extensively used to predict the structural properties of materials and molecules. The main drawback is that its usage grows prohibitively expensive with system size due to the explicit dependence on the underlying electronic structure. Hence, DFT is not used often in global optimization without prescreening using faster, less accurate methods, or other tools that limit the number of evaluations, such as genetic algorithms.^4,5^

Furthermore, identification of the global minima of a given system requires a sufficient exploration of the relevant configurational phase space. This can be expedited with coarse-grained (CG) methods, but these might not fully capture the microscopic details and consequently lead to inaccurate structures and properties due to loss of critical features during the reduction of the detailed atomistic configuration to the CG configuration.^6^

The number of required sampling points can be significantly reduced when employing Gaussian Process (GP) models, as described by Packwood and Hitosugi^7^ and later implemented in the Global Optimization with First-principles Energy Expressions (GOFEE)^8^ and the Bayesian Optimization Structure Search (BOSS) methods,^9,10^ with the latter being considered and applied herein. In this active learning technique, a surrogate model is constructed and iteratively refined through evaluation of an expensive objective function, for instance, the DFT potential energy surface. As a probabilistic method, it assumes the GP posterior mean as the most probable model for the input data, with the corresponding uncertainty described by the posterior variance. The probabilistic nature of the method enables the construction of the surrogate model in fewer data points than that for a corresponding grid search on the full potential energy surface (PES). The utility of BOSS has already been demonstrated for relatively small molecules or systems composed of rigid building blocks with few conformational degrees of freedom, such as the conformer search for cysteine and alanine, the adsorption of an isolated 1*S*-camphor molecule on a Cu(111) surface,^10^ identifying the complex adsorption configurations of tetracyanoethylene (TCNE) on mono- and bilayers on Cu(111),^11^ and the adsorption of Buckminsterfullerene (C_60_) on TiO_2_^9^ to mention a few.

Even with these important achievements, the applicability of BOSS to systems with conformationally highly flexible molecules remains to be demonstrated. The success of BOSS depends on a realistic choice of system variables as the search dimensionality is limited by the sample complexity bottleneck: the underlying dependence of the GP on data set size and number of variables. Consequently, BOSS is currently deemed feasible up to 10–20 variables,^12^ making the system variable choice critical due to the large reduction in dimensionality for the majority of practical organic or biological systems.

Although BOSS has been shown to lessen the computational cost of structure search, the number of necessary configurations to evaluate might still grow too large for DFT, in particular, for flexible molecules with many close-lying conformers. Recently, neural networks have been leveraged to capture the high-dimensional relationship between the structure of a given collection of atoms and the corresponding computed properties, such as energies and forces, using large sets of computed structures. Thus, machine learning interatomic potentials (MLIPs) represent a possible solution when the size of the configurational phase space grows too large for DFT to handle. In principle, MLIPs can be trained at an arbitrarily sophisticated computational level of theory, ranging from DFT to the coupled cluster single-double-triple) level of theory method.^13−16^ By learning the property–structure relationship directly, the need for evaluating the electronic structure is bypassed, significantly accelerating computations.^17^ However, the quality of these potentials depends on the training data, the acquisition of which might be both time-consuming and challenging without a systematic or automated way to select relevant data. To this end, a recent publication demonstrated simultaneous training and exploration of the PES using Gaussian approximation potentials (GAPs).^18^ For our study, we consider the Neural Equivariant Interatomic Potential (NequIP) particularly suitable due to its demonstrated accuracy and data efficiency.^16^

A recent advance for the global optimization of molecular structures is based on metadynamics using the semiempirical extended tight-binding quantum chemistry method GFN2-xTB, as implemented in the Conformer-Rotamer Ensemble Sampling Tool (CREST).^19,20^ Here, traversal of unexplored regions of the PES is enforced by the addition of a biasing potential to already explored regions. The advantage is that CREST can be applied to realistic, high-dimensional phase spaces without having to consider which parts of the system to include as variables in the structure search. This simplifies the use of the tool as no choices about system evolution have to be made. Still, metadynamics rely on a number of low-dimensional collective variables (CVs) to traverse the PES from initial starting configurations, the choice of which is highly sensitive for the end results. Furthermore, its stochastic nature might not always provide the same results, and a large number of data points must be sampled to cover the relevant PES fully. Additionally, there is no publicly available implementation similar to CREST for molecule–surface interfaces, to the best of our knowledge. First and foremost, we will use CREST as a basis of comparison for the global optimization by BOSS for the isolated adsorbates. Additionally, it will be used as an alternative for finding relevant adsorbate conformers should BOSS fail to do so.

As a suitable test for BOSS in the context of flexible adsorbates, we have opted to focus on the adsorption structures of lignocellulosic molecules (LCMs). Lignocellulosic biomass remains an underutilized feedstock for renewable materials. As a chemically heterogeneous composite, it consists of three different kinds of polymers: two carbohydrates, hemicellulose and cellulose, and an aromatic one, lignin.^21^ The first and foremost challenge for the utilization of lignocellulosic biomass is its evolved resistance to degradation, known simply as recalcitrance, rendering component separation a demanding process. A second challenge is to identify the molecular structures of the specific components of the complex heterogeneous material.^22^ Detailed atomistic structural information would not only be useful for the determination of optimal separation methods but also at the same time allow for atom-efficient utilization due to improved book-keeping of present structural moieties. Lignin is a highly cross-linked polymer that is thought to provide plants with their structural rigidity, contributing to significant recalcitrance of the polymer. Cellulose is a polymer made entirely of glucose monomers, while hemicellulose is composed of branched polysaccharides covalently linked to lignin. A major component of hemicellulose is xylan, made up of branched β-1,4-linked-xylose monomers.^23,24^ Already at the monomer level, xylose is a flexible molecule that may undergo a variety of conformational transformations. The most important is ring inversion, characterized by interchanging the positions of the ring substituents between the equatorial and axial positions. The second important transformation is the rotation of the hydroxyl groups as their relative orientations to large extent govern the stability of the molecule.^25^ Traversing the conformational phase space for the ring flip including all ring atoms and the full rotation of all hydroxyl groups implies a 10-dimensional BOSS run for the current internal coordinate-based approach, indicating that the search grows prohibitively large relatively fast.

Concisely, the purpose of this study is to accelerate the investigation of the adsorption structures of hemicellulose building blocks on a surface using Bayesian optimization in conjuncture with MLIPs. The first part of the structure search uses DFT, after which the data used to construct the surrogate model is reused in the training of an MLIP, NequIP. A key assumption is that since the data selected by BOSS is used to find the global minimum structure by rational sampling of the PES, the same data could also be useful in training an interatomic potential to cover related structures. In the second part, we evaluate the suitability of BOSS data as NequIP training data by repeating and further extending the Bayesian optimization structure search with the latter.

## 2 Methods

### 2.1 Workflow

**Scheme 1:**
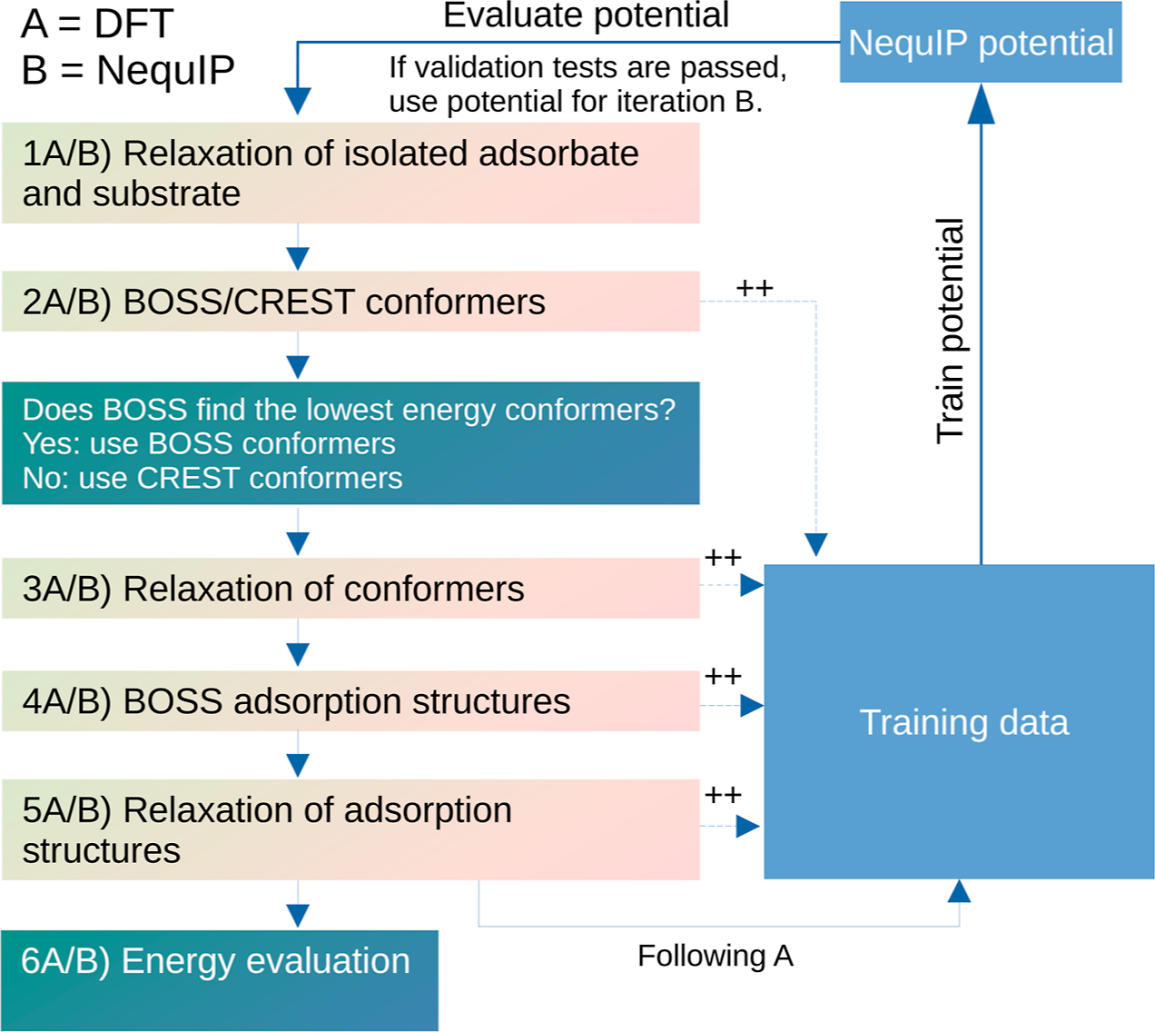
General Workflow

The general workflow of the method described herein is as follows (Scheme 1): (1A) DFT relaxation of isolated adsorbates and substrate, (2A) DFT-based BOSS/CREST conformer analysis, (3A) DFT relaxation of the BOSS/CREST conformers, (4A) DFT-based BOSS adsorption structure analysis using the relaxed BOSS/CREST conformers and substrate as building blocks, and (5A) DFT relaxation of adsorption structures. In the first iteration, BOSS constructs a surrogate model of the DFT PES, simultaneously generating training data for NequIP. Following the training of the interatomic model potentials, the workflow is repeated, but now, a surrogate model is constructed on the PES of the latter. The CREST conformer analysis is not repeated as the conformers are already acquired at this point, but they are relaxed with NequIP if their inclusion is found necessary. Subsequently, the workflow is repeated as follows: (1B) NequIP relaxation of the isolated adsorbates, (2B) NequIP-based BOSS conformer analysis, (3B) NequIP relaxation of the BOSS (or CREST) conformers, (4B) NequIP-based BOSS adsorption structure analysis using the relaxed BOSS (or CREST) conformers and substrate as building blocks, and (5B) NequIP relaxation of adsorption structures. Following each iteration of the workflow, (6A/B) the energies of all local minima of all conformers included in the analysis are compared, providing the global minimum structure. More details on each of the workflow components can be found in the following paragraphs.

### 2.2 Bayesian Optimization Structure Search

The surrogate model of the DFT potential energy surface was learned on the fly starting from the initial five structures and their corresponding DFT energies. The conformational search for β-d-xylose was six-dimensional (6D), including full rotation of the hydroxyl groups and ring-flipping between the two most prominent ring-conformers. In the case of the larger subunit (α-terminated) 1,4-β-d-xylotetraose, the search was 16-dimensional, including rotation of the glycosidic bonds between xylose units. Following the conformational search, the surrogate model was traversed to find the local minima in the model potential energy landscape. Subsequently, the local minima or BOSS-predicted conformers were relaxed with DFT since the reduced dimensionality of the search neglects all degrees of freedom not described by the search variables such as the overall relaxation of the molecule during conformer transformation. In the adsorption structure search, the building block approximation described by Todorović and co-workers was employed, where the rigid conformers were used as building blocks for the adsorption structure, the second one being the surface slab.^9^ During the search, conformers were allowed full translational and rotational freedom (6D) for on-surface motion. Unit cell dimensions were [*a*, *b*] = [2.568 Å, 4.448 Å], defining the bounds for the translational search. For translation in the *z*-direction, the bounds were from 3.0 to 12.0 Å above the surface relative to the geometric center of the adsorbate. The surface symmetry of Cu(111) was leveraged to duplicate symmetrically equivalent structures (2-fold translational, 3-fold rotation at high-symmetry points), effectively growing the data set without any additional computational cost. However, due to the fact that multiple data points will end up in the same local minima, both when minimizing on the BOSS surrogate model PES, as well as during DFT relaxation of the local minima therein, these duplicate points were removed with the Kabsch algorithm.^26^ In addition, we used the energy transformation method as described by Fang et al. to deal with unphysical or high energy configurations that would make the fitting of low energy conformational motion difficult.^27^ Here, a cutoff at 0.5 Å between atoms was used, where the DFT calculation was skipped, and the configuration was assigned a default energy value of 5.0 eV as this represents a reasonable placeholder on the Pauli repulsive part of the approach curve. Furthermore, the high-energy tail of the surrogate model was modified as 1 + log(*E*) eV when the energy was above 1.0 eV. BOSS runs were terminated when the predicted global minimum structure did not change for 100 iterations. A more complete overview of the software implementation of BOSS is given in the original paper ref (9). We also employed BOSS to look for configurations with high energies and forces to help train a more robust MLIP by providing a more complete distribution of the relevant PES in the training data. This was done by running BOSS as usual while minimizing the negative energy of the highest force component in place of the potential energy.

### 2.3 Density Functional Theory

All DFT computations were performed with the PBE + vdW^surf^ method using FHI-aims with light tier-1 basis set defaults on a Γ-point *k*-grid.^28^ Ultimately, the choice of functional was motivated by the property of interest being adsorption structures as this particular van der Waals (vdW)-corrected functional has been shown to provide adsorption energies and heights close to experimental values.^29−32^ It should be noted that the PBE functional might have shortcomings in accurately describing isolated molecules, and its usage should be evaluated case-by-case. Despite this, our computations on xylose reproduced literature results based on a more accurate computational method (second-order Møller–Plesset theory, MP2),^25^ and therefore, we found it acceptable for our application. Both the building block and prediction geometries were relaxed to a force threshold below 0.01 eV/Å, with the charge density convergence threshold (ρ) set to 10^–4^. An orthogonal 6 × 8 × 4 Cu slab was used as the substrate building block for the xylose system, 14 × 16 × 4 for xylotetraose, where the two lowest layers were kept constrained to mimic the behavior of the bulk metal. The slab was constructed using the lattice constant *a* = 3.632 Å from the literature, subsequently relaxed on the PBE + vdW^surf^ level.^33^ The slabs are separated by 60 Å of vacuum in the *z*-direction to avoid interactions. The Atomic Simulation Environment (ASE)^34^ was used to manipulate, create, and visualize both conformer and adsorption structures for the computations. POV-ray^35^ was used to create images of the structures.

### 2.4 Neural Equivariant Interatomic Potentials

In addition, we also applied the NequIP framework with high data efficiency to train interatomic potentials based on the DFT data we generated, thus enabling faster energy evaluation of structures. In the original NequIP paper,^16^ it was demonstrated that the inclusion of equivariance leads to significant improvements in the accuracy of an MLIP as seen through lower force and energy mean average errors. In fact, NequIP without equivariance performed similarly to other potentials such as FCHL19, UNiTE, GAP, ANI, and ACE, even being outclassed by some of these for several of the molecules tested. However, adding equivariance boosted the performance of NequIP far beyond the mentioned MLIPs. Furthermore, the increased data efficiency of NequIP was demonstrated by highlighting a particular instance, where NequIP trained on a small set of 100 entries performed on par with a Behler-Parrinello Neural Network (BPNN) trained on 1303 entries.

The important hyperparameters for the training (more details in the data set repository: DOI:10.5281/zenodo.10202927) used by all models were as follows: interaction blocks num_layers = 4, the multiplicity of features num_feature = 32, cutoff radius *r*_max_ = 3.5 Å, and the maximum rotation order *l*_max_ = 2. The batch size was 10 for the training data set. The mean average error (MAE) loss function was given as the sum of total energy and forces loss terms with a ratio of 1:1, which was minimized to optimize the neural network using Adam optimizer with a learning rate of 0.005 and an exponential moving average decay of 0.99. The trained interatomic potentials for the xylose and Cu(111) system were further used to predict energies and simulate dynamic trajectories for adsorbate conformers and adsorption structures through integration into the ASE using the NequIP calculator with the Broyden–Fletcher–Goldfarb–Shanno optimizer.^36−39^ Typically, the force thresholds for relaxation were kept the same as with DFT, with the exception of the BOSS run for xylotetraose, where the value was slightly elevated to 0.03 eV/Å to facilitate the complete exploration of structures. We found a minimal difference in these structures with a higher value during testing. The training was repeated on a number of different training sets produced during the BOSS procedure, containing both isolated and surface-adsorbed LCMs.

### 2.5 Conformer-Rotamer Ensemble Sampling Tool

The use of CREST in this study had two purposes; it was either used to evaluate the quality of the BOSS conformational analysis or to replace BOSS were it unable to find the lowest energy conformers for the system at hand. If both methods resulted in similar conformational ensembles, we used the BOSS conformers for the subsequent adsorption structure search. We used the sampling tool as implemented in the xtb package. The first step in the CREST algorithm relaxes the geometries with GFN2-xTB. Subsequently, the length of the metadynamics simulation required to cover the relevant conformational phase space was estimated by calculating the total flexibility measure for the molecules. The total flexibility measure was calculated to be 0.17 and 0.33 for xylose and xylotetraose, respectively, resulting in total metadynamics simulation times of 70 and 336 ps, respectively.

## 3 Results and Discussion

### 3.1 Global Conformer Minimum Search with BOSS (DFT) and CREST

**Figure 1:**
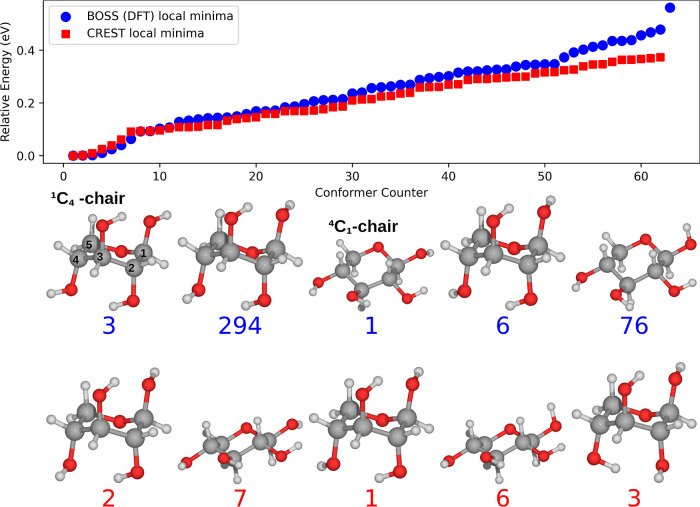
Relative energies of the PBE + vdW^surf^ relaxed BOSS (500 data points were used to construct the surrogate model) and CREST-predicted conformers of β-d-xylose. The five most stable conformers from each method are displayed in the order of increasing energy from left to right. The energy ordering of the predictions equals the shown conformer indices.

The main results of the global xylose conformer minimum search are summarized in Figure 1. Both BOSS and CREST results follow a similar overall energy distribution, indicating that they both sample and capture similar structural features of the xylose conformers. The close energies of several conformers indicate that the conformational PES is relatively flat. Furthermore, both methods provide a similar global minima structure after DFT relaxation, the ^1^*C*_4_ chair configuration (BOSS local minima 3, 294, and 6), only differing slightly in the rotation of the OH-groups. Qualitative agreement is obtained between our study and a combined experimental and computational study by Peña et al.^25^ Therein, the most stable conformer is the ^4^*C*_1_ chair, equivalent to BOSS conformer 1 (and CREST 7) in Figure 1, while their second (^1^*C*_4_ chair) and third (also ^1^*C*_4_ chair) lowest conformers are identical to BOSS 6 (and CREST 1) and CREST locmin 3, respectively. The importance of the intramolecular hydrogen-bonding network was emphasized, stabilizing the isolated xylose molecule through cooperativity effects. The latter was used to rationalize why the ^4^*C*_1_ chair was found to be the most stable, seeing as all four OH-groups of this conformer are involved in the network. At this point, too much weight should not be placed on the energy order of the conformers but rather on the ability of the methods to locate conformers. The agreement between our analysis and that of Peña and co-workers demonstrates that both BOSS and CREST are able to locate the relevant xylose conformers. However, when BOSS is used, it should be pointed out that transitioning between the ^1^*C*_4_ and ^4^*C*_1_ chair configurations does not capture all stable monosaccharide conformers by default. For instance, glucose and mannose display stable boat (B) and skewed boat (S) conformers outside the conformational phase space that is sampled during the specific ^1^*C*_4_ to ^4^*C*_1_ transition, while the conformational phase space of xylose is more completely mapped out in this reduced phase space.^40−42^ Taking this into consideration, we suggest a more complete traversal of the different ring conformers when investigating other monosaccharides, for instance, using Cremer and Pople puckering coordinates.^43,44^

**Figure 2:**
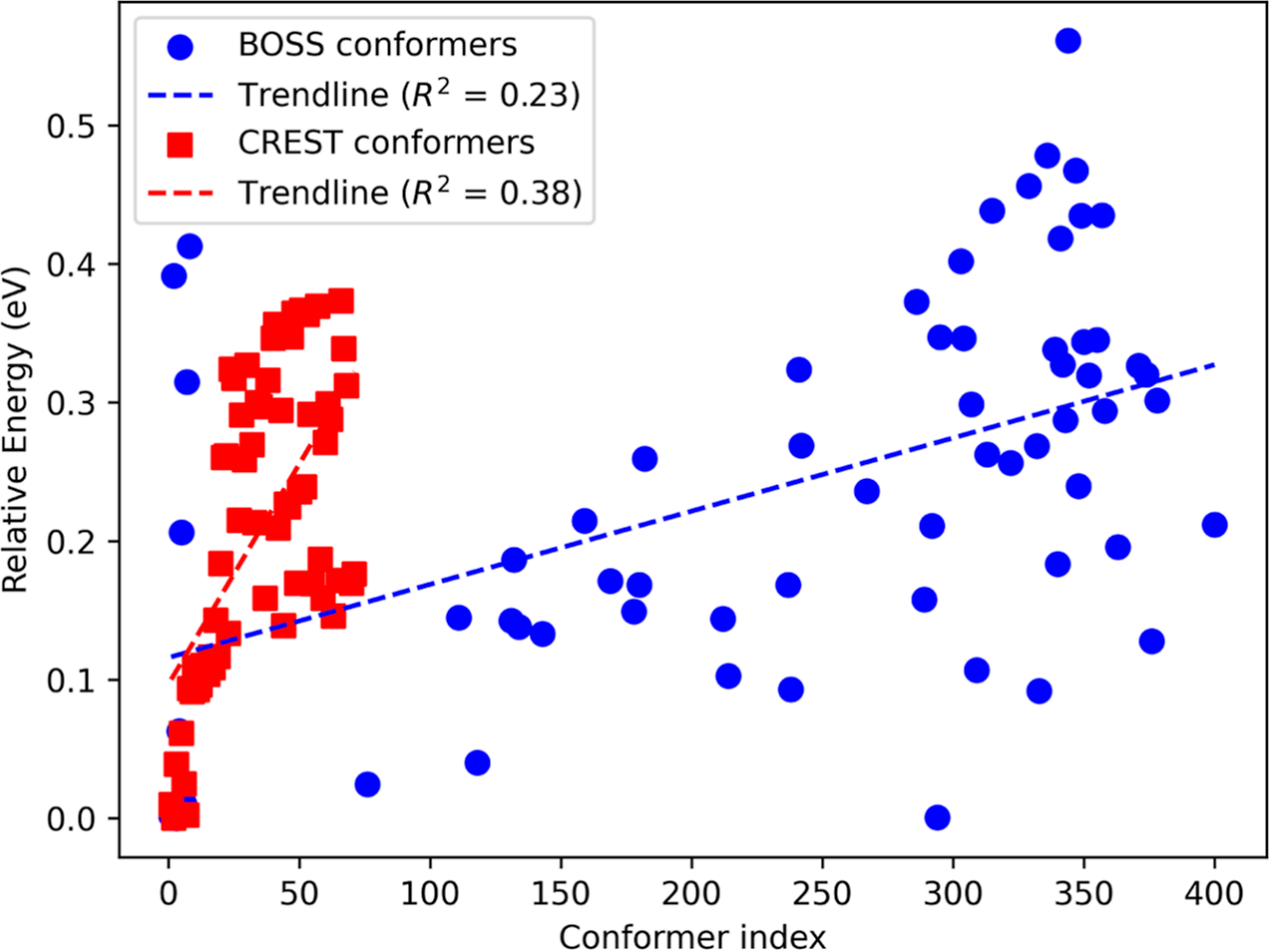
Relative PBE + vdW^surf^ energies of relaxed xylose conformers as predicted by BOSS/CREST according to their predicted energy order, denoted by the conformer index corresponding to BOSS/CREST predictions. Note that the index is not representative of the final number of unique DFT-relaxed conformers and that the energies shown are relative to individual BOSS and CREST set minima.

The energies of all PBE + vdW^surf^ relaxed BOSS and CREST predictions are displayed in Figure 2, simultaneously showing the predicted energetic order of the two methods, where a lower conformer index corresponds to a lower prediction energy. Although the DFT energies increase with the predicted energies, the correlations (*R*^2^-values) are low. This can be partly attributed to the conformational relaxation of the predicted local minima during the DFT relaxation procedure, highlighting the necessity of the latter when using this method. We believe that rest of the discrepancy might be attributed to the effects arising from uncertainty in the surrogate model PES. Generally, the ability of BOSS to predict the DFT energies of conformers is connected to how closely the reduced-dimensional representation follows the conformational transformation on the DFT PES. In other words, the predicted order will consequently be sensitive to the choice of initial relaxed DFT structure acting as the starting point since it is this specific structure that is altered in a reduced-dimensional phase space during Bayesian inference. For this particular case, the structure was the ^4^*C*_1_ chair, and therefore, it is not entirely unexpected that the equivalent BOSS local minimum structure 1 has the lowest predicted energy. To investigate the effects of the initial structure on the predicted order, we repeated our analysis with a ^1^*C*_4_ chair as the initial structure (Supporting Information S2.2). Although the predicted and final conformer orderings change slightly, similar conformers are found even when starting with a different initial structure.

**Figure 3:**
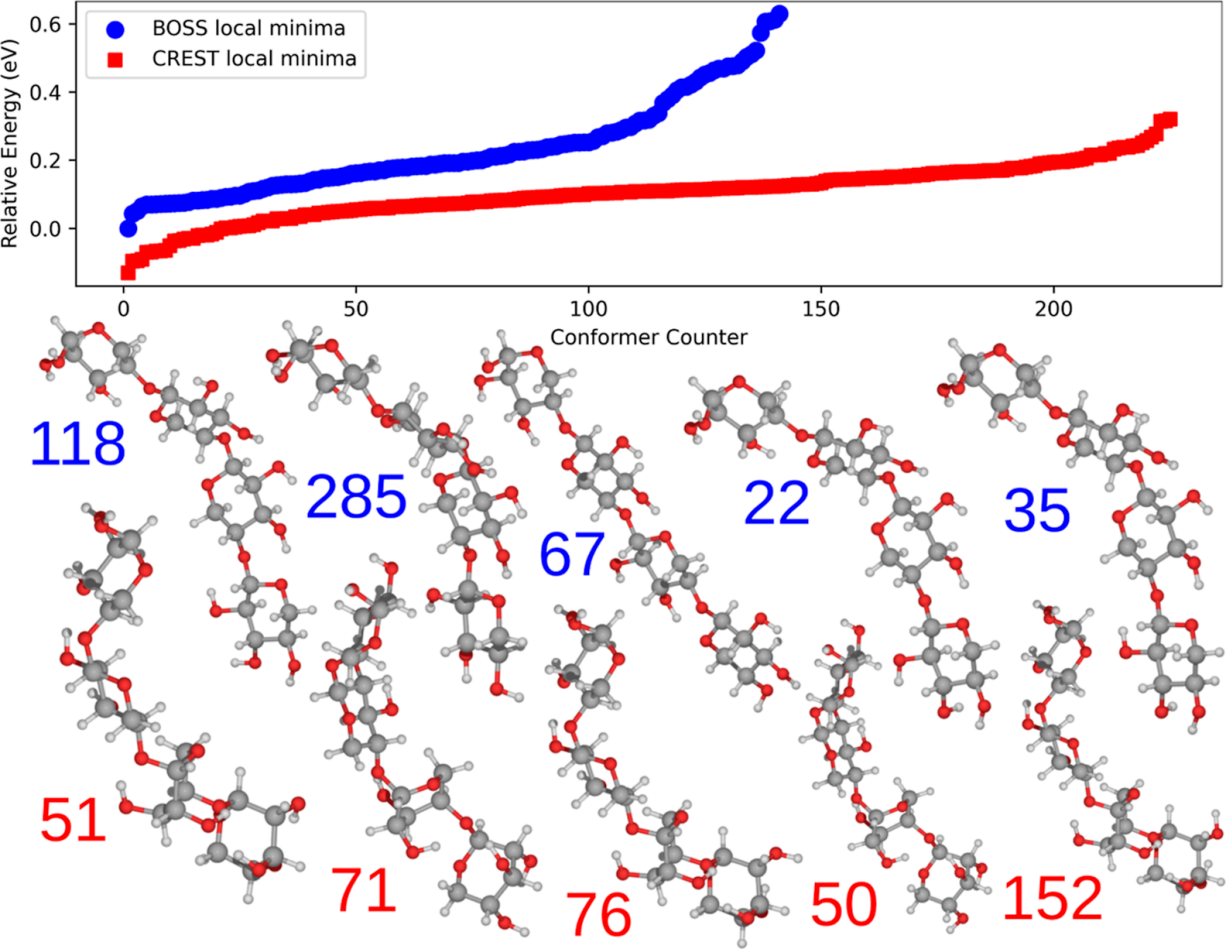
Relative energies of the PBE + vdW^surf^ relaxed BOSS (909 data points were used to construct the surrogate model) and CREST-predicted conformers of 1,4-β-d-xylotetraose. The five most stable conformers from each method are displayed in order of increasing energy from left to right. The energy ordering of the predictions equals the shown conformer indices.

In contrast to the xylose conformer search, BOSS and CREST display more dissimilarities in the energy distributions for xylotetraose conformers, as shown in Figure 3. The most striking result is that CREST arrives at a lower energy for the global minimum than does BOSS with the current choice of degrees of freedom, including only rotations of the glycosidic bonds and hydroxyl groups. Inspection of the five lowest energy structures reveals how the lower energy is attained with a mixture of ^4^*C*_1_ and ^1^*C*_4_ chair configurations on the constituent xylose monomers of the xylotetraose chain than that with only ^4^*C*_1_, as is the case with BOSS. Unfortunately, including the ring-flip for the search would imply 24 degrees of freedom, which is intractable for the underlying GP without first bypassing the sample complexity bottleneck. In this regard, recent work has demonstrated the feasibility of higher dimensionality (*D* > 20), achieved by mapping the high-dimensional problem to a low-dimensional feature space.^12,45^ The already implemented Bayesian optimization routine could be slightly augmented with neural networks for learning a response surface in low-dimensional feature space (encoder). This would then be followed by acquisition function minimization as already implemented but now in feature space. Finally, the full objective function can be evaluated after projecting the low-dimensional feature into the high-dimensional original parameter space using a decoder. Successful implementation would also enable simultaneous conformation and adsorption structure determination, simplifying the process by negating the need for two separate steps. In this light, we consider this to be a promising direction for future implementation of the methodology described herein.

Although a few xylotetraose conformers have been reported, no comprehensive studies have been published on the gas-phase conformers, to the best of our knowledge. For instance, a study on the enzymatic cleavage of arabinoxylans mentions two conformers: the first where all the xylose units attain the ^4^*C*_1_ chair configuration, while the second has one unit in a skewed boat ^2^*S*_0_ configuration.^46^ While the relative energies of the two conformers were not explicitly stated, the highest occupied molecular orbital–lowest unoccupied molecular orbital gap was found to be larger for the ^4^*C*_1_ than for the ^2^*S*_0_ conformer, suggesting at least higher kinetic stability for the former. Another study on the same cleavage mechanism reports the same two conformers as well as an inverted boat conformer ^2,5^*B*.^47^ The computed relative energies of the ^4^*C*_1_ and ^2^*S*_0_ conformers were later reported by the same group, placing the chair configuration 0.2 eV below the skewed boat.^48^ The aforementioned results are in line with experimental studies, where many carbohydrates indeed show a preference for the ^4^*C*_1_ chair.^49^ It would therefore be interesting if mixed-ring xylotetraose conformers were actually more stable than the ^4^*C*_1_ chair counterpart, in particular, on the surface.

To investigate the role of the terminating group on the preferred ring configurations, we applied CREST on both α- and β-terminated xylotetraose. Interestingly, the global minimum conformer for β-terminated xylotetraose is one, where three out of four xylose units are in ^1^*C*_4_ configurations and one in ^2^*S*_0_ (Supporting Information S3.1), which is 0.18 eV higher in energy than the corresponding α-terminated global minimum. The change in ring-conformer preference suggests that termination does play a role in the overall distribution of ring conformers in xylotetraose.

**Figure 4:**
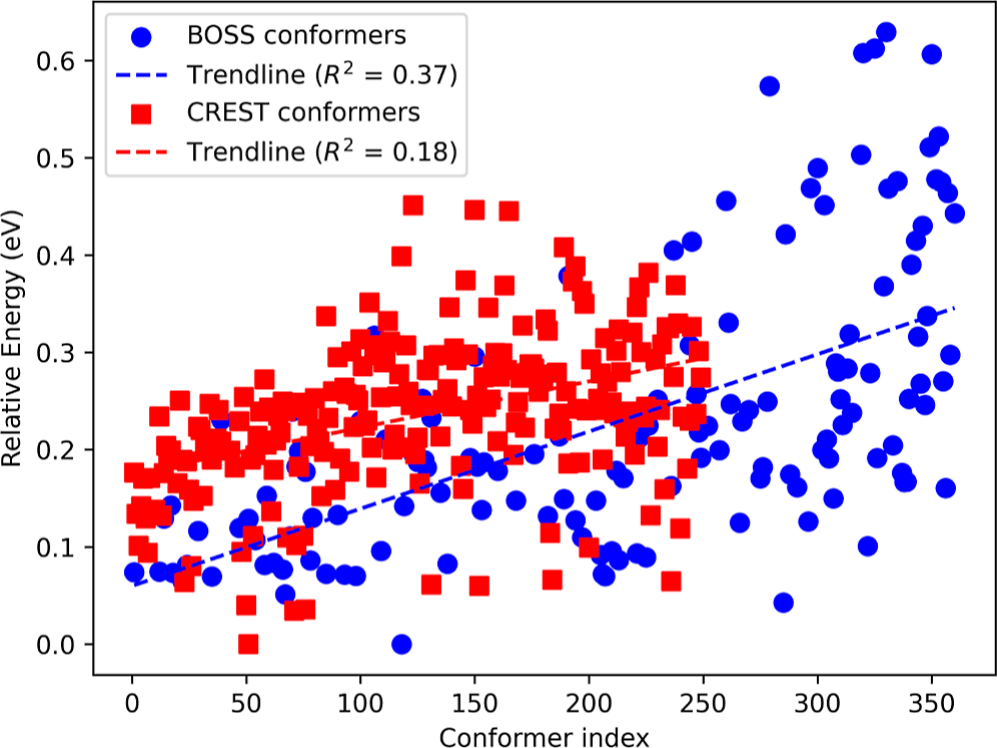
Relative PBE + vdW^surf^ energies of relaxed xylotetraose conformers as predicted by BOSS/CREST according to their predicted energy order, denoted by the conformer index corresponding to BOSS/CREST predictions. Note that the index is not representative of the final number of unique DFT-relaxed conformers and that the energies shown are relative to individual BOSS and CREST set minima.

Similar to the situation for the xylose conformers, a weak trend of increasing DFT energies with the BOSS/CREST predicted counterparts emerges, as shown in Figure 4. We believe that this is insufficient for the purpose of leveraging any possible correlations between the energies of isolated molecules and their adsorption structures to accelerate the search since DFT relaxation is evidently necessary to determine the relative energies of the latter.

### 3.2 Global Adsorption Structure Minimum Search with BOSS (DFT)

To find the global minimum of a molecular adsorbate–substrate system, one must either sample a representative conformer ensemble with rotational and translational freedom on the surface (within the building block approximation) or conduct the conformational search on-surface with rotational and variational degrees of freedom included. Inclusion of all conformers for the full global minimum search was found to be too computationally expensive when using DFT, even though this brute-force approach represents a way to ensure the identification of the global adsorption structure minimum. Although the BOSS part alone might have been feasible for this system, the subsequent relaxation of local minima requires disproportionately many computational hours. Consequently, a representative set of the conformational ensemble was selected for the adsorption structure search with DFT. This set included both of the most stable chair forms, ^4^*C*_1_ and ^1^*C*_4_, as well as less stable boat conformers, such as the ^1,4^*B* chair. The orientation of the hydrogen bonds was deemed less important as the rotation of the hydroxyl groups displays a lower barrier than ring-inversion on the surrogate model PES (Supporting Information S2.1).

**Figure 5:**
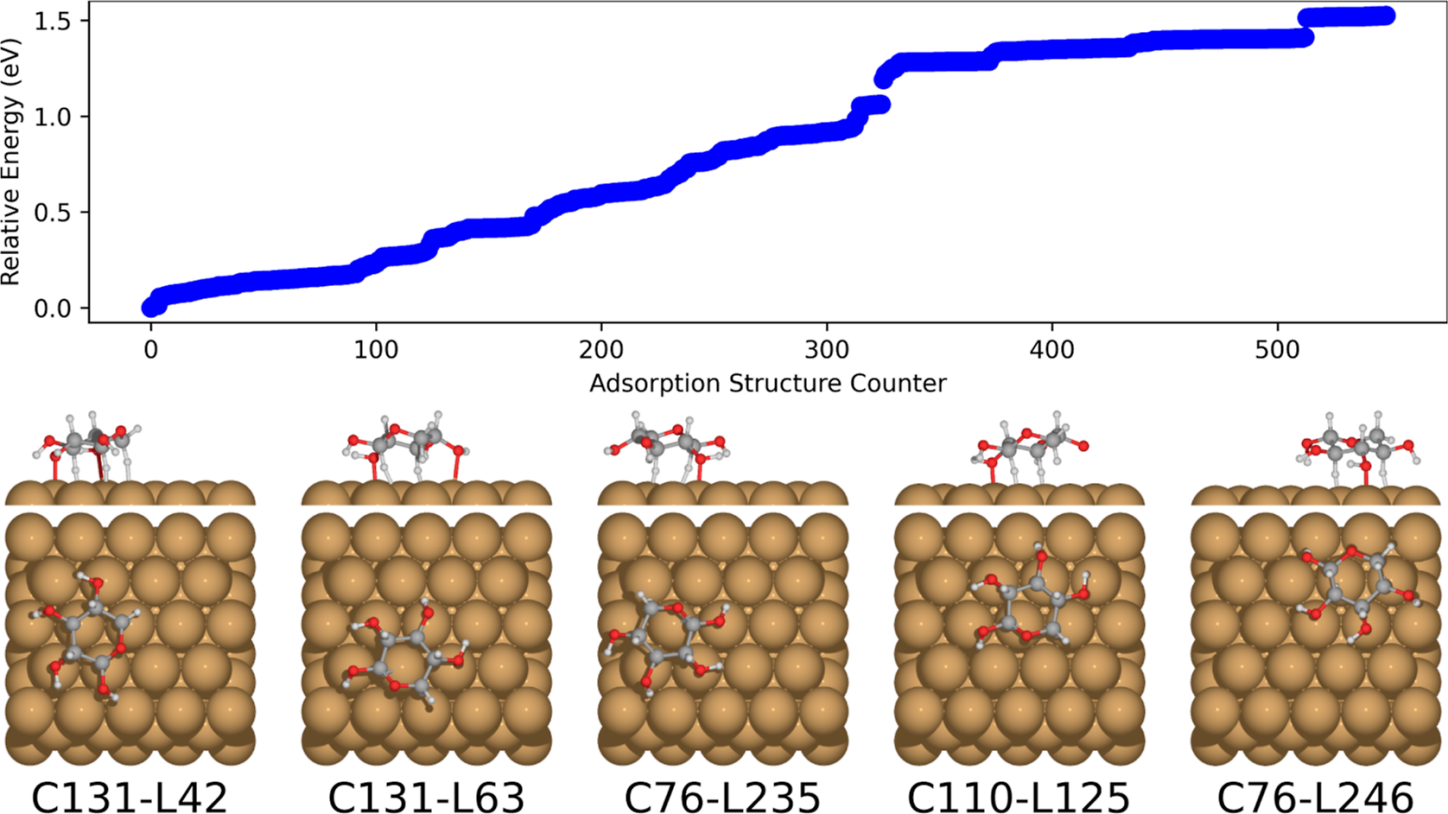
Relative energies of the PBE + vdW^surf^ relaxed adsorption structures of a representative selection of β-d-xylose conformers (76, 110, 111, 118, 131, 182, and 397) on Cu(111), as predicted by BOSS. Typically, around 1000 data points were used to construct the adsorption structure surrogate models. The five most stable adsorption structures are displayed in order of increasing energy from left to right. The BOSS energy ordering equals the local minimum index.

The five lowest energy adsorption structures are all found to be the ^4^*C*_1_ chair, as shown in Figure 5. The most stable position on the surface has the *C*_1_ hydroxyl and the ring oxygen atoms in bridging positions, while the surface-oriented axial hydrogen atoms of the xylose ring are close to a top position on the Cu(111) surface.

**Figure 6:**
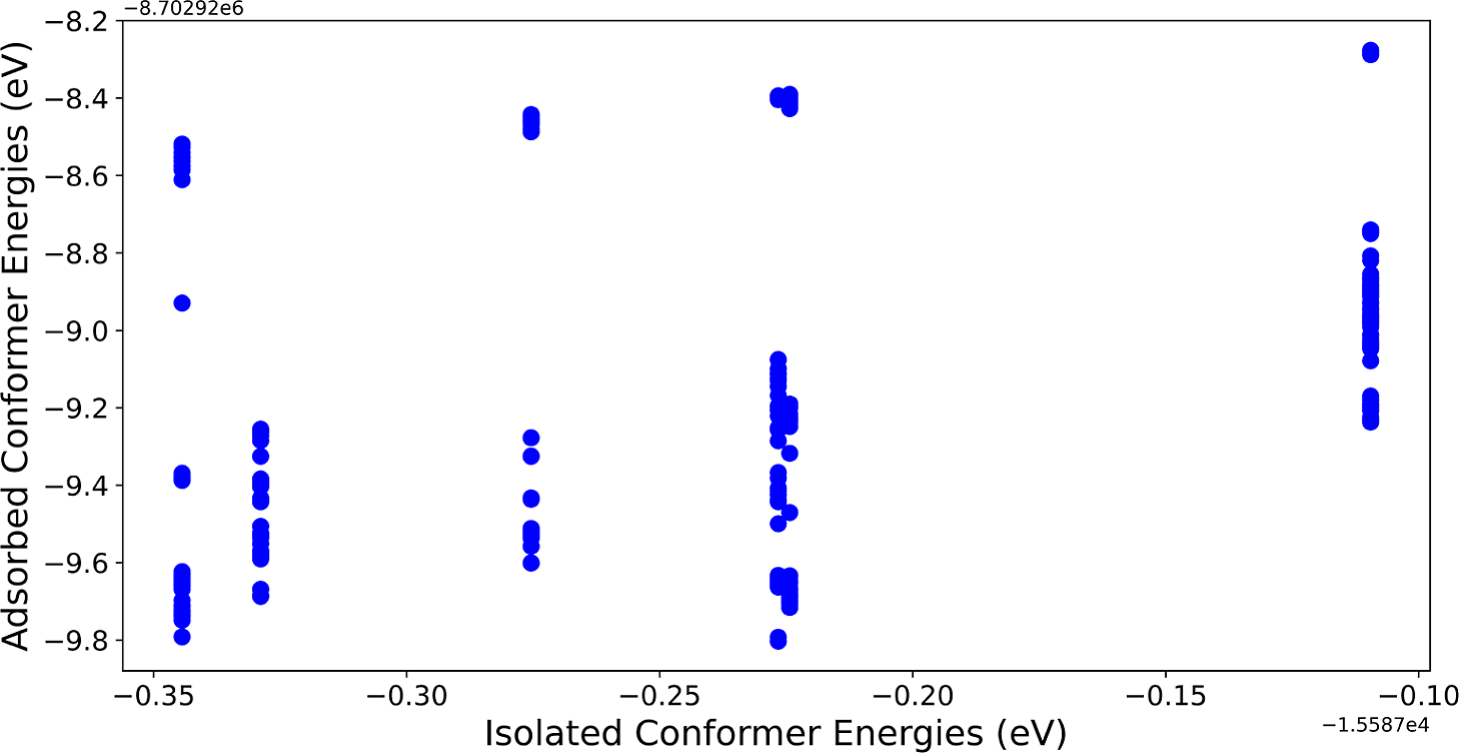
Absolute energies of the DFT relaxed β-d-xylose adsorption structures predicted by BOSS with DFT, arranged by conformer stability.

The prediction of global adsorption minima would be greatly simplified if a general trend emerged when comparing the energies of isolated conformers to those of their most stable adsorption structures, as illustrated in Figure 6. Unfortunately, even though there seems to be a slight upward trend indicating that more stable conformers also lead to more stable adsorbate–substrate systems, the data is too sparse to allow for confident determination. Especially since the lowest-energy adsorption structure of each individual conformer is more randomly dispersed than the corresponding conformer average.

**Figure 7:**
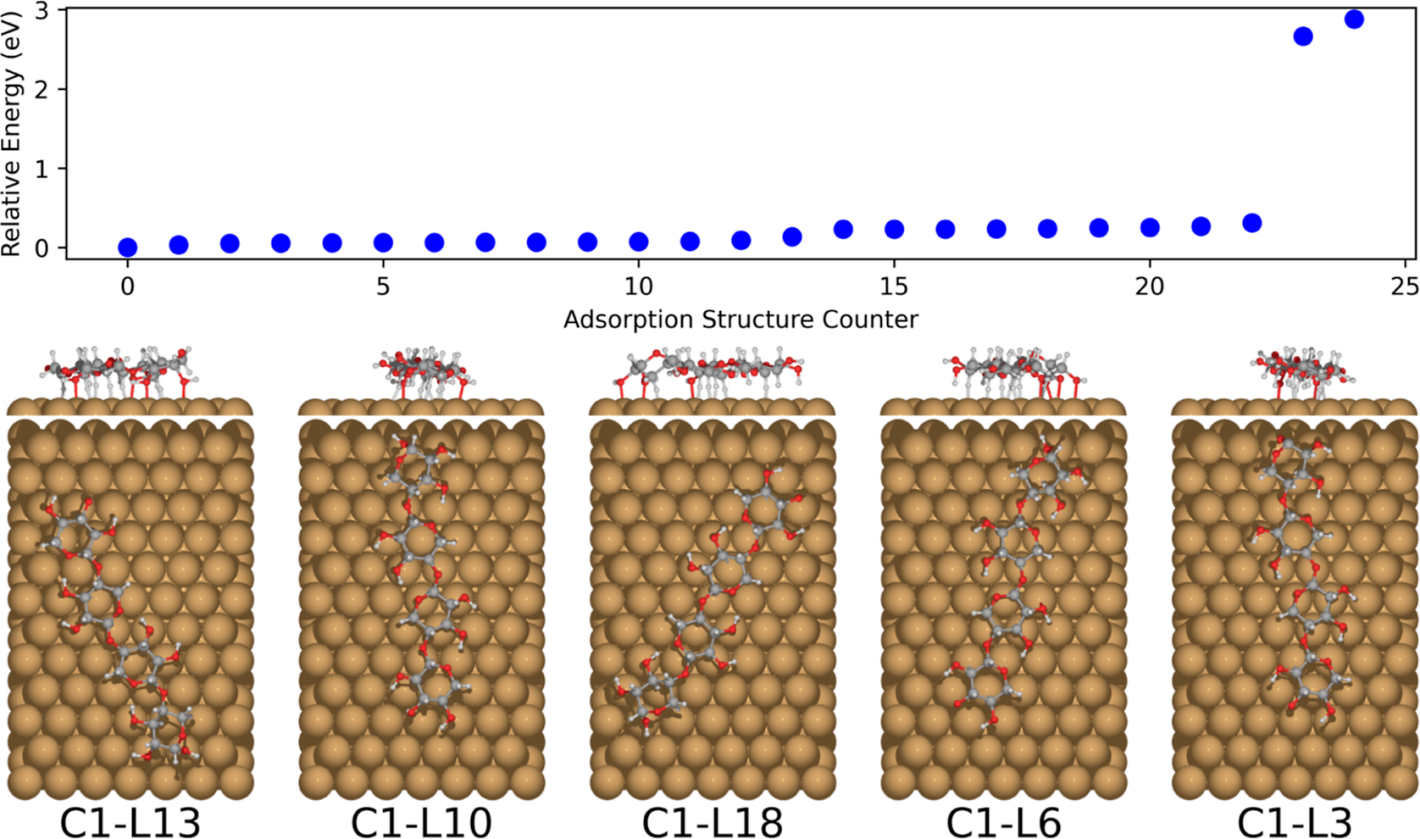
Relative energies of the PBE + vdW^surf^ relaxed adsorption structures of two β-d-xylotetraose conformers (1 and 32) on Cu(111) as predicted by BOSS (1000 data points were used to construct the surrogate model of conformer 1, 477 points for conformer 32). The five most stable adsorption structures are displayed in order of increasing energy from left to right. The BOSS energy ordering is equal to the locmin index.

The results of the DFT based global adsorption minimum search for xylotetraose are summarized in Figure 7, which we admit includes very few structures to allow a confident identification of the global minimum. Also, due to the minimal amount of structures that we found feasible to relax, the included conformers were chosen based on chemical intuition rather than systematically within the workflow. The relaxation of local minima extracted from the BOSS surrogate model was typically much slower than the xylose adsorption structures. Furthermore, due to the fact that we wanted to model isolated on-surface adsorbates, 10 Å of free space in each direction along the surface was needed, and the substrate slab is therefore much larger than that for xylose on copper (212 vs 967 total atoms). Hence, using DFT to look for the global adsorption minimum structure for xylotetraose was found to be intractable due to the limited number of configurations that could be possibly visited and relaxed. However, from the data that we have, we can note that the most stable adsorption structure has the glycosidic and ring oxygen atoms in or close to bridging positions, which is characteristic of the xylose global minimum as well. Thus, the interaction between the two different LCMs and the copper surface bears some important similarities, which we might be able to leverage to save resources on the NequIP training.

The degree of proximity between the BOSS surrogate model and CREST structures to DFT-relaxed structures is approximated by the average number of DFT relaxation steps as depicted in Table 1. We supplement this approximation with the corresponding RMSD values between relaxed and unrelaxed (predicted) structures. Typically, BOSS local minima require almost three times as many relaxation steps to get to the minima as CREST. Meanwhile, xylotetraose uses almost twice as many steps as xylose, which is not too surprising considering the size difference. Similar conclusions can be made by inspecting the corresponding RMSD-values. An important implication is that the choice of BOSS degrees of freedom becomes even more critical for relaxation with growing system size, especially for flexible species. For the adsorption configurations, we notice that both xylose and xylotetraose require similar number of relaxation steps. This could indicate that relaxation on the surface does not scale as drastically with the size of the adsorbate as does the conformational dynamics, perhaps due to the surface interaction becoming more important than conformational relaxation when the adsorbate approaches a surface.

**Table 1:** Average Number of DFT Relaxation Steps (*N*_steps_) from BOSS/CREST-Predicted Local Minima, Root-Mean-Square Deviations (RMSD in Ångstroms), and General Computational Resource Usage

	Xylose^a^	Xylotetraose^b^	Xylose on Cu^c^	Xylotetraose on Cu^d^
*N*_steps_ BOSS to DFT	56	106	70	74
*N*_steps_ CREST to DFT	21	34		
RMSD BOSS to DFT	0.23	0.59	0.28	0.16
RMSD CREST to DFT	0.08	0.10		
DFT CPU usage (time (s)/step × CPU)	0.004	0.028	0.636	3.868
BOSS total wall time (s)	15571^e^	388800^f^	604240^g^	777600^g^
CREST total wall time (s)	140^e^	11527^e^		

a 63 BOSS conformers.

b 141 BOSS conformers.

c 7 of 63 conformers.

d 2 of 141 conformers.

e 48 CPUs.

f 256 CPUs.

g 512 CPUs.

At this point, it should be noted that CREST is much faster computationally than BOSS due to the underlying semiempirical method for the energies and forces, yet arrives at conformers closer to the DFT relaxed counterparts than BOSS. We surmise this to be due to the BOSS conformers being determined in the reduced-dimensional phase space with BOSS, while the CREST conformers are relaxed on the GFN2-xTB level during the sampling process. However, if the structure of the system of interest is governed by effects not captured by the semiempirical method, CREST would not necessarily be able to capture the relevant conformational dynamics, while in principle, BOSS can be tuned to use a suitable quantum chemistry method.

### 3.3 NequIP Validation Tests

**Figure 8:**
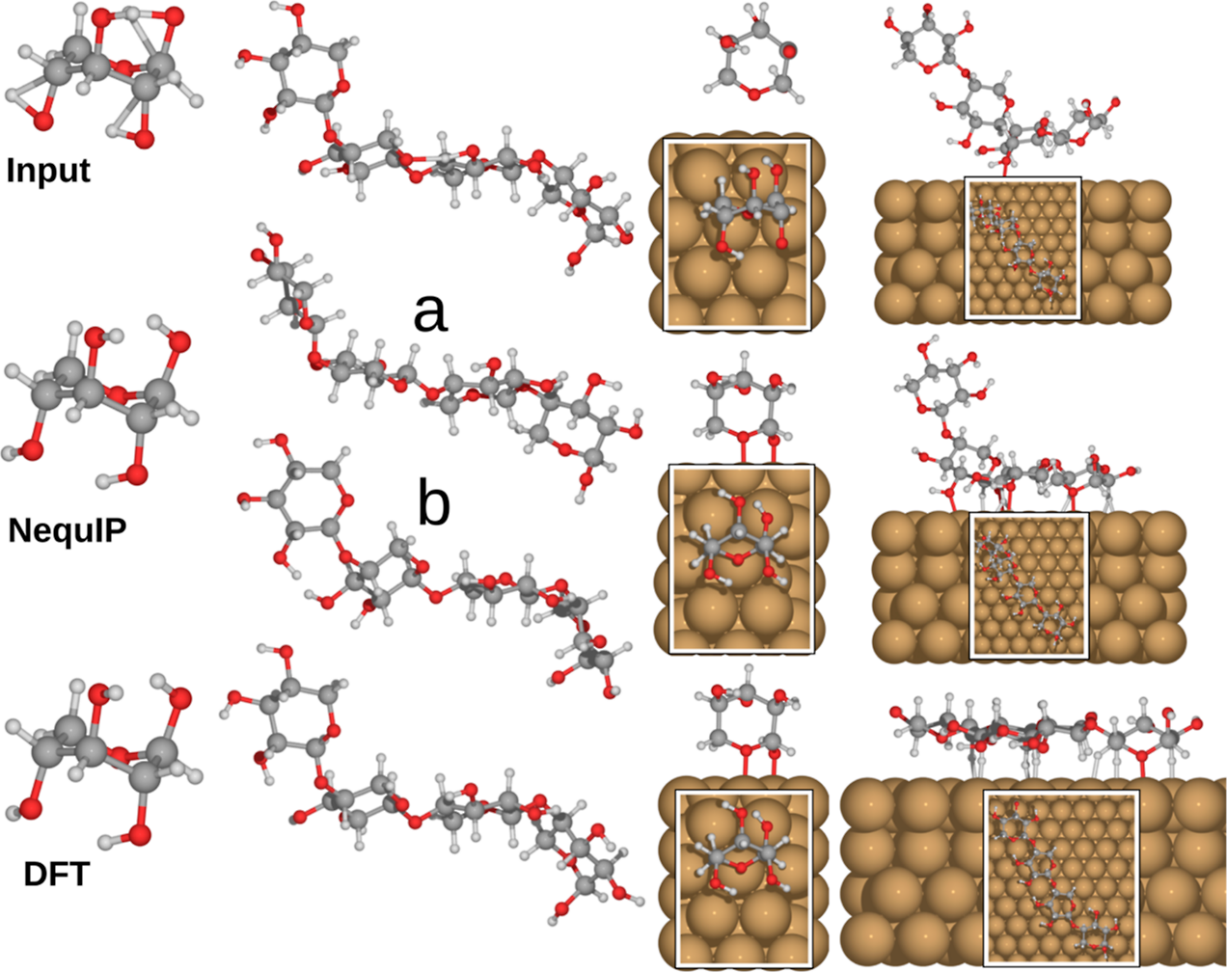
PBE + vdW^surf^ vs NequIP relaxation of xylose- and xylotetraose adsorbates and adsorption structures. The shown examples were all relaxed with NequIP potential 3 from Table 2, with the exception of b, which was relaxed using potential 8 from Table 2.

**Figure 9:**
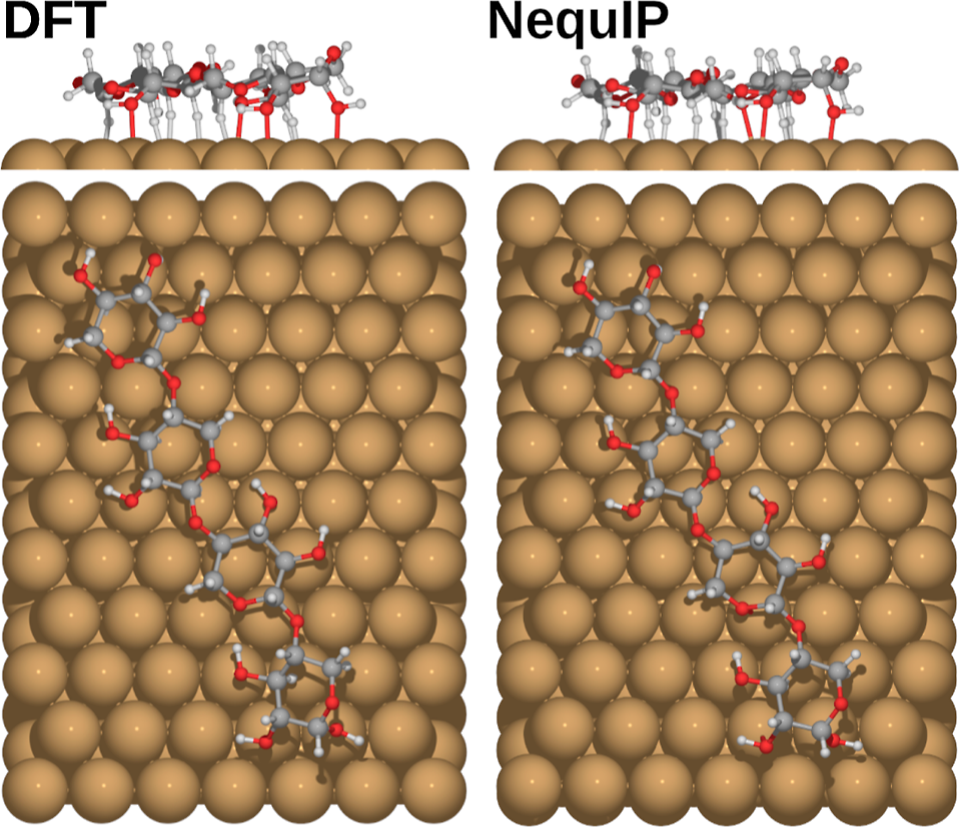
Comparison of DFT (PBE + vdW^surf^) and NequIP-relaxed xylotetraose global minimum structure as determined by BOSS (DFT).

To accelerate the structure search further than by Bayesian optimization alone, we trained NequIP using data from our BOSS workflow ran with DFT. We wanted to determine if the training data could affect the reliability and efficiency of the potential, hence multiple potentials were trained for this purpose. In the assessment and validation of the training, we focused on how well the potential in question reproduced the DFT structures and at the very least the relative energies since the potential would subsequently be used to run BOSS with NequIP as a faster substitute for the former. A simple test for how well the potential would perform in an applied setting was therefore to use it for the relaxation of adsorbates and adsorption structures and subsequently compare these with the corresponding DFT structures. A sample of the validation tests are illustrated in Figure 8 and closer details on the training data are shown in Table 2. Models that included high-energy configurations in the training are denoted by fmax in the name, i.e., potentials 3 and 5. Validation tests for the remainder of the potentials are shown in the Supporting Information S1.2. Relaxation of a distorted xylose conformer with elongated and rotated bonds results in identical conformers for DFT and NequIP. This is also the case for rotated xylose conformer 397 on the Cu(111) surface. For xylotetraose, the agreement with the DFT relaxation is not particularly good, which likely stems from the fact that the potential did not include any xylotetraose data in the training. Therefore, we repeated the training with xylotetraose conformers and the few surface adsorption configurations we had, and the resulting potential (8) yields a structure (b) much closer to the DFT counterpart. This demonstrates how a potential can be improved by including more data, in particular, data containing more diverse structures. For xylotetraose on the surface, the difference is much more distinct, where one end of the chain fails to relax on the surface. This failure can perhaps be expected due to the small amount of training data for this particular adsorbate. When relaxing this structure with potential 8, the whole chain manages to relax onto the surface (Figure S5, Supporting Information S1.2) Nonetheless, we surmised that the xylotetraose surface interaction should be rather similar to that of xylose, which made up the most of our NequIP training data. In support of our assumption, we relaxed the DFT-based global minimum (C1-L13, Figure 7) using potential 3, which led to minimal rearrangement, as shown in Figure 9. Through our tests, we also observed complete failure for several of the potentials, even for the simple case of isolated conformers, where the system would explode. This was typical for potential 1, which included the whole relaxation data set without any curated selection of the training data. We selected the potential to be used with BOSS based on how well the NequIP relaxations followed DFT overall, taking also into account the amount and diversity of data needed for training. A nice balance was found for potential number 3. The training for this potential included only relaxation data for a selection of xylose adsorbates on the copper surface, and no conformation data. Still, acceptable performance was observed even for the latter type in our tests. Furthermore, the potential in question did not fail completely in any of our test cases, whereas many had issues with surface relaxation. We suspect this to be due to the surface atoms being more or less in identical positions in the whole training set, which does not provide any information about forces and energies when the surface deviates more than slightly from the training configuration. A quick solution to avoid issues with the surface was to simply constrain it during relaxation, found to be a valid approximation since relaxation with a fixed surface generally provided similar adsorption heights and geometries as without—when using potentials having no issues in this regard. Thus, we might surmise that the interaction of the adsorbate with the surface is generally well described in the training data. Hence, potential 3 was used to obtain all NequIP results presented hereafter, unless specified otherwise.

**Table 2:** NequIP Training Data Details

Name (no.)	*N*_train/val_	Type	Epochs	Training energy and force errors (eV/*N*_atom_, eV/Å)
Cu 11574 (1)	9000/2000	adsorption structures	475	0.0007/0.002
Cu 76 111 (2)	9000/1700	adsorption structures	581	0.0008/0.006
Cu 76 111 fmax (3)	9000/140	adsorption structures	1459	0.0004/0.001
Mix 76 111 118 (4)	7000/2000	adsorption structures + conformers	1006	0.0003/0.001
Mix 76 111 118 fmax (5)	7000/2000	adsorption structures + conformers	1024	0.0004/0.002
Xylo model 1 (6)	2200/292	adsorption structures	918	0.0004/0.001
Xylo model 2 (7)	10500/1500	adsorption structures	3683	0.0007/0.001
Mix xylotetraose CREST (8)	3000/487	adsorption structures + conformers	1173	0.002/0.007

In an attempt to assess the quality of our trained potentials, we analyzed their training and validation losses (Supporting Information S1.1). We were particularly interested in the occurrence of overfitting, which would limit the applicability of the potentials for out-of-distribution data. A telltale sign of overfitting is an increasing validation loss with training,^50,51^ which is observed in the case of potential 4. However, this is not a large concern as the deployed model is based on the best training model, taking validation loss into account. A validation loss much larger than training loss could also indicate a model unable to generalize to new data, which is the case for potentials 1, 2, 3, and 5. Despite this, we note that potential 3 still performs fairly well on unseen data as exemplified by the relaxation of xylotetraose, both as an isolated molecule and on the surface. Ultimately, we find that the inclusion of high-energy configurations makes for a more robust potential, as can be seen from the training metrics and through the validation tests.

### 3.4 Global Conformer Minimum Search with BOSS (NequIP)

**Figure 10:**
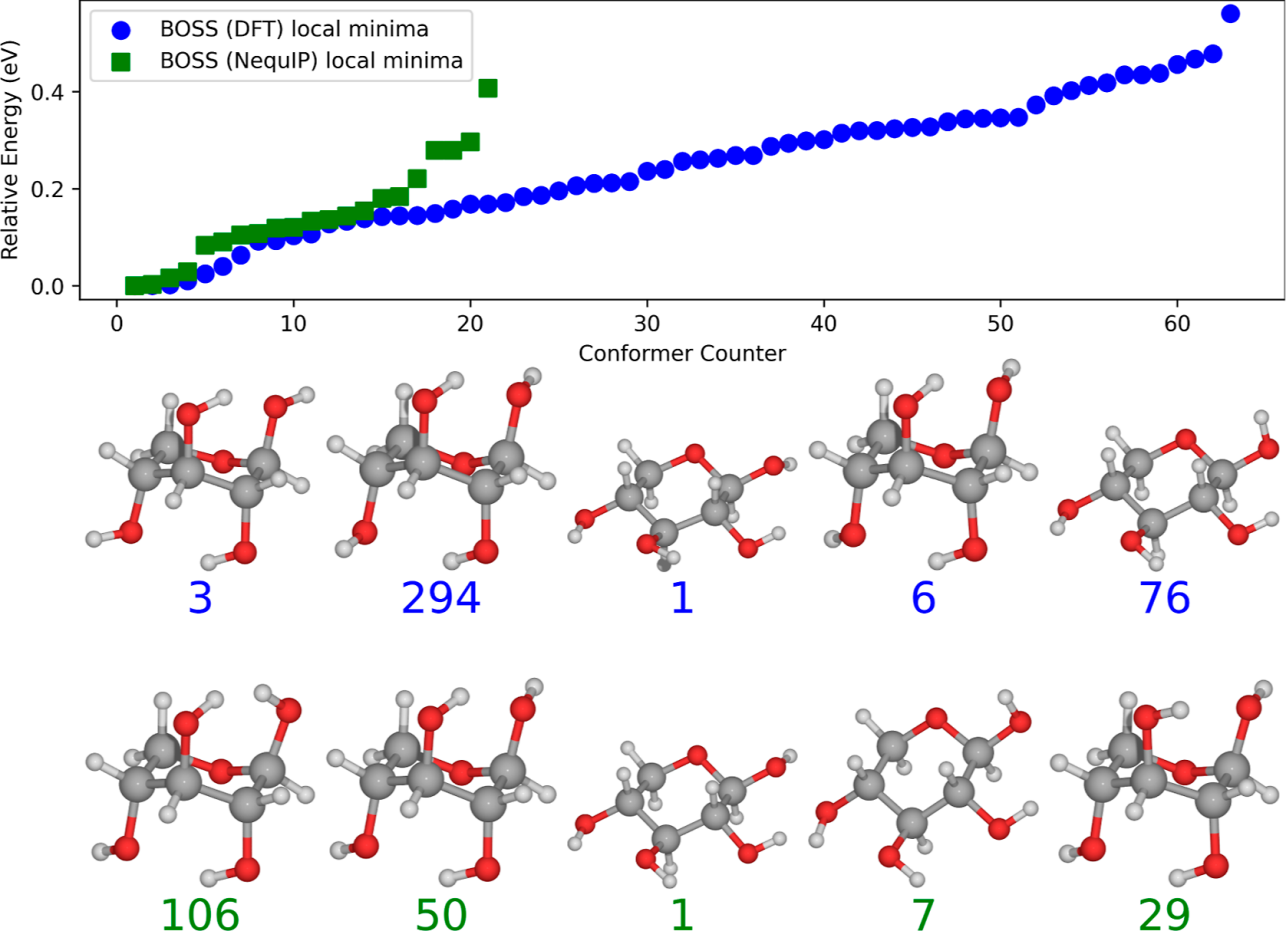
Relative NequIP energies of the relaxed β-d-xylose conformers predicted by BOSS with the NequIP PES as the target for the surrogate model (based on 377 data points to construct the surrogate model) in comparison to the DFT results. The five most stable adsorption structures are displayed in order of increasing energy from left to right. The BOSS energy ordering is equal to the locmin index.

Following the NequIP training, we used BOSS again to identify the local minima but with the trained NequIP PES landscape as the target for the surrogate model. The idea was that this would allow us to evaluate the adsorption structures of the full conformational ensemble since energy-evaluation is much more rapid with NequIP than DFT. From inspection of the results in Figure 10, fair agreement with DFT-based BOSS is achieved. However, the predicted most stable structure (106) has two hydroxyl hydrogens in close proximity, a configuration that would exhibit significant steric strain. Other than this obvious error, the other conformers are in line with DFT. It should be pointed out that the NequIP potential used here was trained only on surface adsorption structures, meaning that the training data do not include data from the conformational analysis. In this respect, the potential captures the conformational dynamics quite well, even with sparse data. On the other hand, this might explain the relatively few conformers found. When this strained structure was relaxed with another NequIP potential (8), the hydroxyl groups rotated to a more realistic orientation, pointing away from the other hydroxyl group. Potential 8 was trained on data from both the BOSS conformer and adsorption structure analyses as well as high-energy data.

### 3.5 Global Adsorption Structure Minimum Search with BOSS (NequIP)

**Figure 11:**
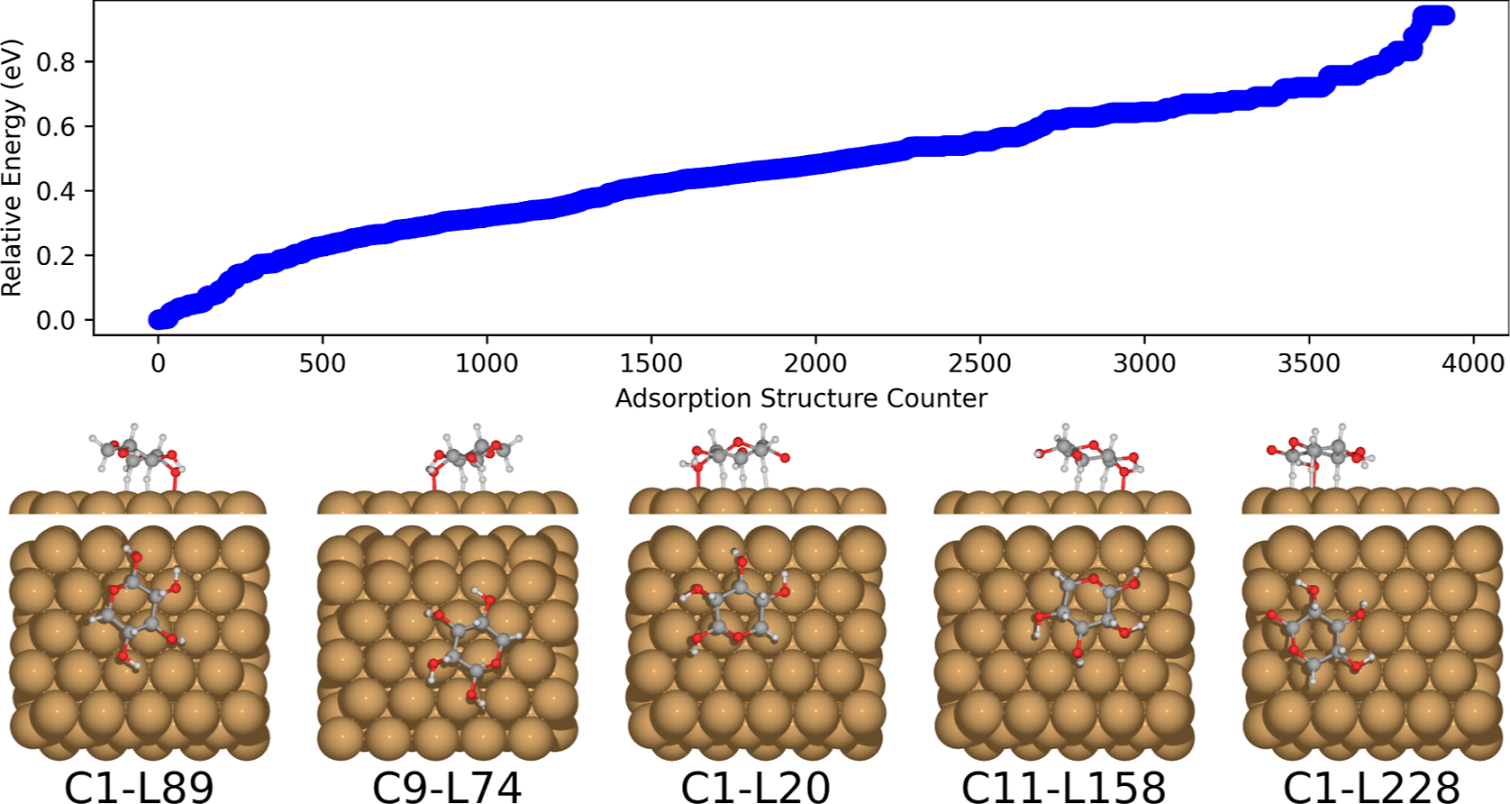
Relative NequIP energies of the relaxed β-d-xylose adsorption structures on Cu(111) predicted by BOSS with the NequIP PES as target for the surrogate model. Based on 1000 < data points to construct the surrogate model. The five most stable adsorption structures are displayed in order of increasing energy from left to right. The BOSS energy ordering is equal to the locmin index.

As seen in Figure 11, the lowest energy adsorption configurations resemble the ones obtained from the BOSS run with DFT. When comparing NequIP to the DFT based search, we note that we get about ten times as many adsorption configurations in a fraction of the time, demonstrating the acceleration of the structure search. To test how well the MLIP replicates the DFT geometries, we ran relaxation using PBE + vdW^surf^ on the lowest energy NequIP adsorption structures. The DFT relaxations produced negligible changes in the geometries (20 > relaxation steps, average RMSD from DFT relaxed structure was 0.05 Å), meaning that, at least for the five lowest energy configurations, NequIP reproduces the DFT structures well (Supporting Information S4.1). Even the energies display a similar trend of the structures being nearly isoenergetic. Overall, the structure search with BOSS and NequIP combined is able to find the same minima as DFT-based BOSS, and the former even finds an equivalent structure to the DFT global minima in C1-L20. Still, these structures are mostly within or close to the training distribution, and the real test for assessing the out-of-distribution performance of NequIP is the adsorption structure search for xylotetraose. Success in this area implies that neural network potentials can be used reliably as predictive tools for these systems, which is one of the most important characteristics of DFT.

**Figure 12:**
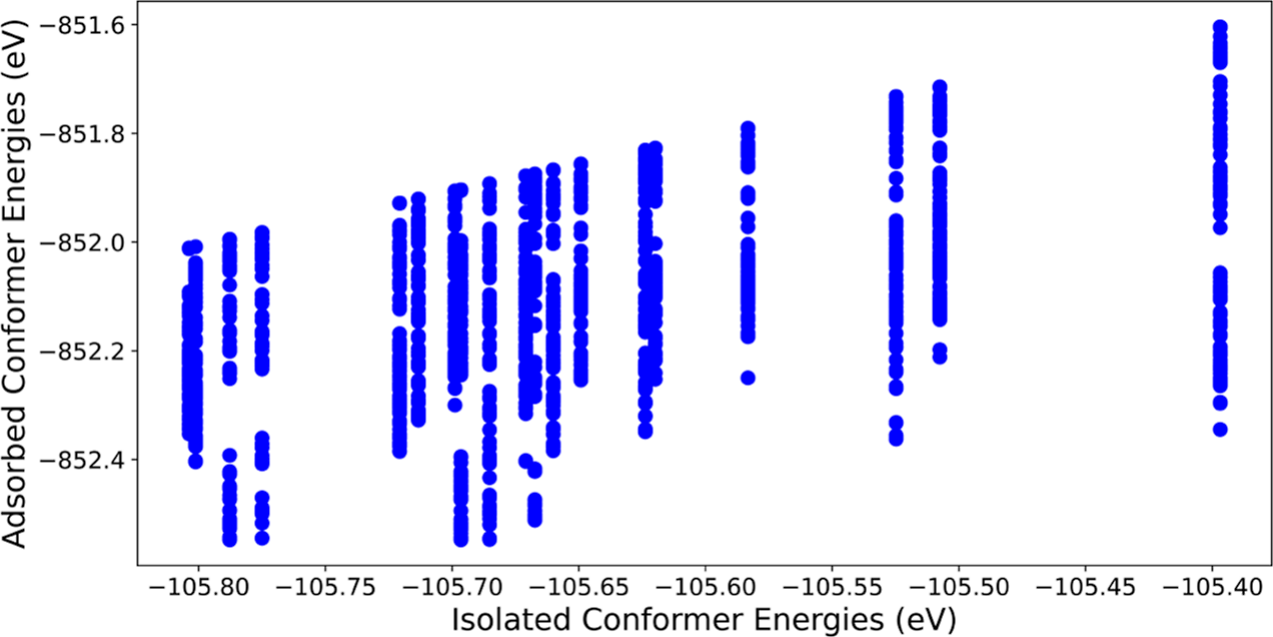
Absolute energies of the NequIP relaxed β-d-xylose adsorption structures predicted by BOSS with NequIP, arranged by conformer stability.

As already noted in the earlier subsection on xylose adsorption structures during the discussion on isolated adsorbate energies and adsorption structure energies, there is no unambiguous correlation between the two, with the exception of the highest adsorption energy of each isolated conformer, as this corresponds to the energy of the adsorbate in a dissociated state (see Figure 12). This illustrates how the properties of a molecule that govern its stability are not innately linked to the way that it interacts with the surface. However, it could be emphasized that the global adsorption minima belongs to a conformer in the lower energy range, and thus it might be possible to discard some of the higher energy ones before BOSS runs to save time and resources.

Although infeasible with DFT, NequIP enabled us to both sample and relax a large number (10^5^) of xylotetraose adsorption structures due to much faster energy evaluation at 16 s/step on 1 CPU compared to 1408 s/step on 512 CPUs (the latter amounting to 300 years of CPU time for the same number of structures). The computational resources spent on the two LCMs investigated in this study are summarized in Tables 3 and 4. When comparing DFT using 512 CPUs to NequIP using only one CPU, also accounting for the training of the MLIP, the latter is about 40 times faster. Meanwhile, the savings in computational resources are more accurately illustrated through estimated billing units. Here, we made estimates of the billing units required to sample the same number of structures using both methods, considering how the DFT structural ensemble is much smaller than that of NequIP. Furthermore, the estimate includes the resources spent on obtaining the training data. The computational resources spent on acquiring all of the training data for the various potentials herein–around 12 000 DFT data points–amount to 107 days of computational time (1 027 200 billing units), including the DFT relaxations and the BOSS procedure used to find the initial configurations. Accounting for the training data acquisition and training, we estimate that BOSS (DFT) requires ten times as many billing units as that for BOSS (NequIP) in the xylose case, while the whole analysis with both LCMs makes the latter method 1500 times cheaper in terms of billing hours. At this point. it should be mentioned that a reliable NequIP potential (potential 8) for this particular system was also acquired using 3000 data points, a third of the training data for the potential (potential 3) used for the analysis described here. Even smaller data sets ranging from a few hundred to a little over a thousand data entries were described in the original NequIP-paper.^16^

**Table 3:** β-d-Xylose Total Computational Resource Usage by Method

	BOSS (DFT)^a^ wall time (days)	BOSS (NequIP) wall time (days)
conformers	0.20	0.06
adsorption structures	1119.00^b^	23.37^c^
MLIP training		2.95
total	1119.20	26.38 [133.38]^d^
billing units	10 742 400	495 [1 027 695]^d^

a Estimated for the same number of structure evaluations as done with NequIP.

b 512 CPUs, including training data acquisition.

c 1 CPU.

d Including training data acquisition.

**Table 4:** β-d-Xylotetraose Total Computational Resource Usage by Method

	BOSS (DFT)^a^ wall time (days)	BOSS (NequIP) wall time (days)
conformers	5.12	0.43
adsorption structures	171 082.00^b^	4166.67^c^
MLIP training		
total	171 087.12	4167.10
billing units	1 642 387 200	78 133

a Estimated for the same number of structure evaluations as done with NequIP.

b 512 CPUs.

c 1 CPU.

**Figure 13:**
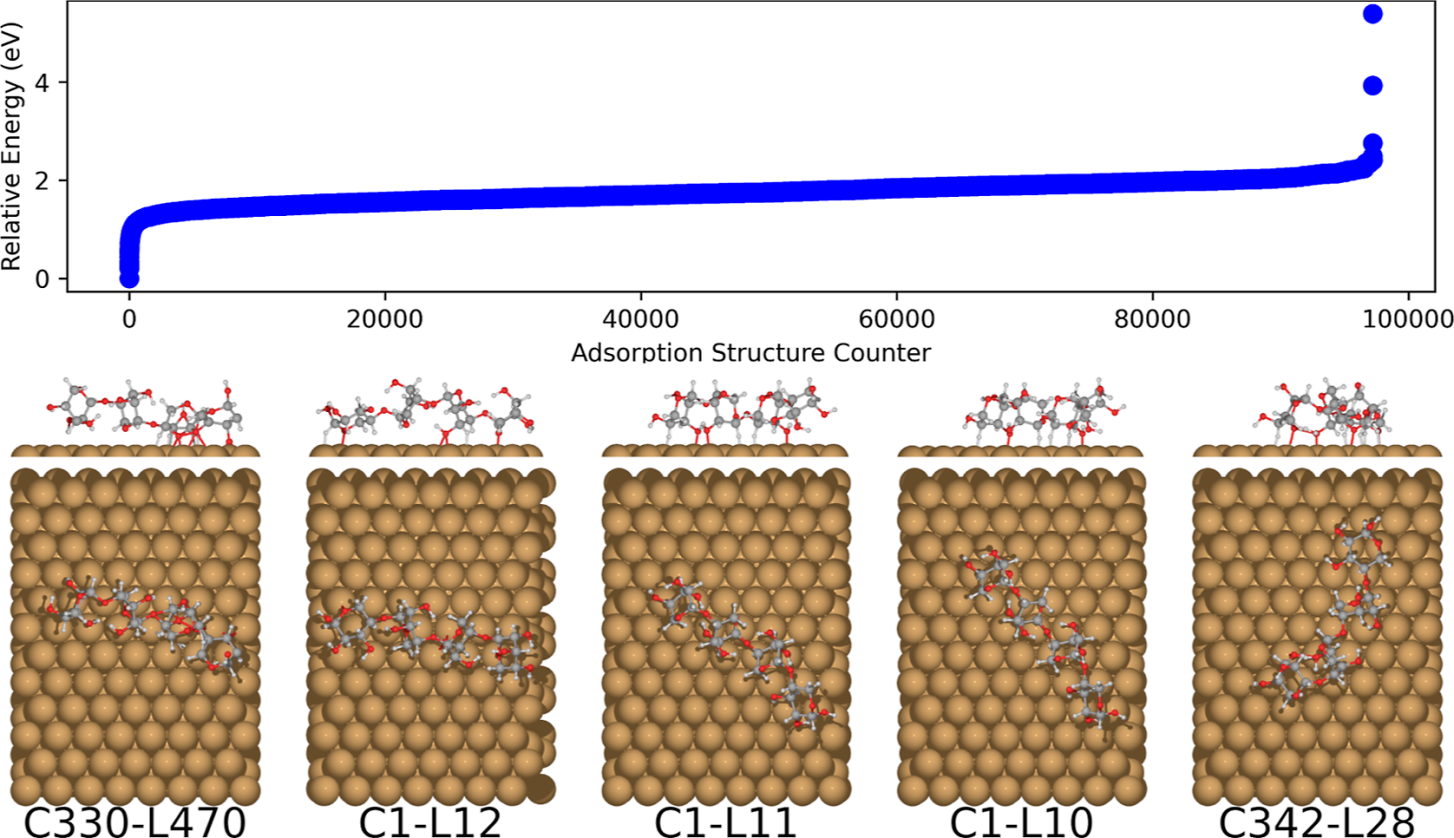
Relative NequIP energies of the relaxed β-d-xylotetraose adsorption structures on Cu(111) predicted by BOSS with the NequIP PES as a target for the surrogate model. The five most stable adsorption structures are displayed in order of increasing energy from left to right. The BOSS energy ordering is equal to the locmin index.

**Figure 14:**
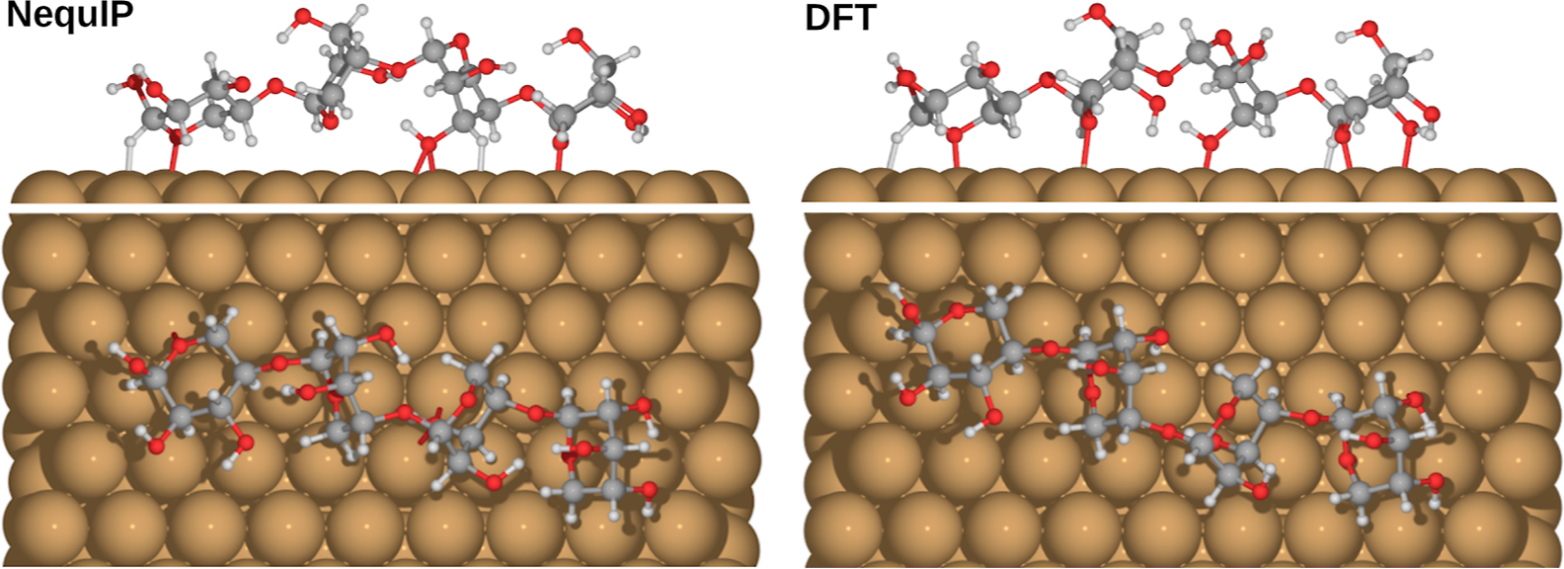
DFT (PBE + vdW^surf^) relaxation of the xylotetraose adsorption structure C1-L12 determined by BOSS (NequIP).

Since the conformer search indicated that CREST arrived at the lowest energy conformers, these were used as adsorbates in the adsorption structure search instead of the BOSS-determined conformers. The results of this sampling are displayed in Figure 13. We note how the predicted global minimum is clearly unphysical with distorted features, and we discard it in the following discussion. We surmise that NequIP has predicted this as a more stable structure due to a more close proximity of the molecule to the surface. Another clear error is in structure C342-L28, in which two hydroxyl oxygens are both covalently bonded (0.97 Å) to the same hydrogen. The rest of the displayed structures are physically more realistic at first inspection. Putting this to the test, the lowest-energy structures (except C330-L470) were relaxed with DFT (Supporting Information 4.2). In general, the xylotetraose chain elongates slightly on the surface during relaxation, indicating that the potential falls somewhat short of accurately capturing the interactions between units. Still, considering the rather low interaction cutoff distance of 3.5 Å and the fact that there were no xylotetraose data in the training set, the agreement between the methods is promising. Moreover, the adsorption height typically changes by only 0.1 Å during DFT relaxation of the NequIP structures, even with the changes in the adsorbate structures (average RMSD between NequIP and DFT for the four lowest structures is 0.18 Å). The performance of NequIP for adsorption structures is well illustrated with a side-by-side comparison with DFT, as shown in Figure 14 with the C1-L12 adsorption structure. Here, the xylotetraose chain is elongated by 0.6 Å during relaxation, and the adsorption height of the molecule measured relative to the closest atom to the surface is reduced by 0.1 Å. The positions of the adsorbate on the surface change to reflect the elongation, and the geometric center of atoms moves by about 1.2 Å. Hence, the overall adsorption structure is preserved better than the adsorbate position on the surface.

Now, when the relative DFT and NequIP energies of the lowest structures are compared, the energy order is maintained even with large variations in absolute differences. This indicates that the method could be used to identify the global minimum structure. However, the more stable CREST conformers do not automatically lead to lower adsorption structures than those from the DFT-based BOSS run. In fact, the global adsorption minimum as determined herein is the C1-L13 structure with BOSS conformer 1, being 0.5 eV lower in energy than the global minimum derived from CREST conformers, C1-L12. This means xylotetraose with all rings in the ^4^*C*_1_ configuration attains the most stable adsorption structure. The fact that NequIP missed this structure is due to the assumption that the CREST conformers would be the most relevant in order to find the global minimum, and these did not include the same conformers as determined by BOSS. However, when relaxing the BOSS-derived C1-L13 structure with the NequIP potential, C1-L12 is slightly lower in energy than C1-L13, meaning that our NequIP potential would fail to assign the latter as the global minimum, even if the BOSS-derived conformers were included. Ultimately, we realize that while the DFT energy ordering is more accurate, experimental data on this system would help determine the minimum and would thus be the best way to assess the methodology herein. It should also be noted that a more accurate method would account for vibrational contributions to the energies, and to that end, we wish to test the applicability of MLIPs in the computation of vibrational frequencies in the future.

## 4 Conclusions

We have demonstrated how the combined use of BOSS and NequIP accelerates and enables the search for global adsorption minimum structures of highly flexible lignocellulosic molecules. This is most evident when size becomes a limiting factor for DFT, yet NequIP provides structures of similar fidelity to the latter. While BOSS was found to be somewhat restricted for the conformer analysis, supplementation of external conformer search tools alleviates this, here exemplified with CREST. The machine learning interatomic potential NequIP performs well in replicating the DFT global minima of the xylose system. Furthermore, the performance of the out-of-distribution xylotetraose system is at the very least promising, which is where the savings of the computational resources put in to train an interatomic potential should be the largest, considering the cost of DFT for evaluating a comparable number of structures. Our findings suggest that to achieve an interatomic potential with some degree of generalization, the training set should contain enough of the structural features or interactions important for the systems to which one is trying to generalize, such as adsorbate–substrate interactions at varying orientations and distances, as well as a sufficiently diverse conformational ensemble. An unanswered question emerges from this study: Could the NequIP-based structure search be used to find the global minimum of a system even if the DFT training data do not include it? To answer this, we might have to expand upon our analysis by repeating the BOSS conformer analysis with a NequIP potential trained on a more complete conformer ensemble for the xylose system and include both BOSS and CREST conformers in the xylotetraose case. However, in the end, a more reliable evaluation of the methods would be done together with suitable experimental structural determination methods. In future work, we therefore aim to investigate the suitability of the methodology described herein in aiding the characterization of three-dimensional lignocellulosic molecules by atomic force microscopy. Another important aspiration of ours is to accelerate the search further by restricting the number of needed data evaluations and structural relaxations by constraining the phase space based on plausible structures deduced from experimental images.

## Supporting Information Available

Statistics on the NequIP training, more complete overview of the NequIP validation tests, details on BOSS for xylose including the surrogate model for the predicted global minimum, xylose conformer analysis with ^1^*C*_4_-initial structure, the effect of anomerism on the most stable xylotetraose conformer determined by CREST, DFT relaxations of xylose adsorption structures predicted by BOSS (NequIP), and DFT relaxations of xylotetraose adsorption structures predicted by BOSS (NequIP) In addition, all structures with corresponding energies, as well as the NequIP training configuration files will be made available in Zenodo (10.5281/zenodo.10202927) (PDF)
