# Supplementary material for: Accelerated Lignocellulosic Molecule Adsorption Structure Determination
Source: J Chem Theory Comput. 2024 Feb 26;20(5):2297–312. doi: 10.1021/acs.jctc.3c01292 (PMC10939001; doi:10.1021/acs.jctc.3c01292)
Supplement: Supplementary file 1 — ct3c01292_si_001.pdf [file ct3c01292_si_001.pdf]

Supporting Information: Accelerated lignocellulosic molecule adsorption structure determination

Joakim S. Jestilä,[*,†] Nian Wu,[†] Fabio Priante,[†] and Adam S. Foster[*,†,‡]

†*Department of Applied Physics, Aalto University, 00076 Aalto, Espoo, Finland*

‡*Nano Life Science Institute (WPI-NanoLSI), Kanazawa University, Kanazawa 920-1192, Japan*

E-mail: joakim.jestila@aalto.fi; adam.foster@aalto.fi

1 NequIP

1.1 Training metrics

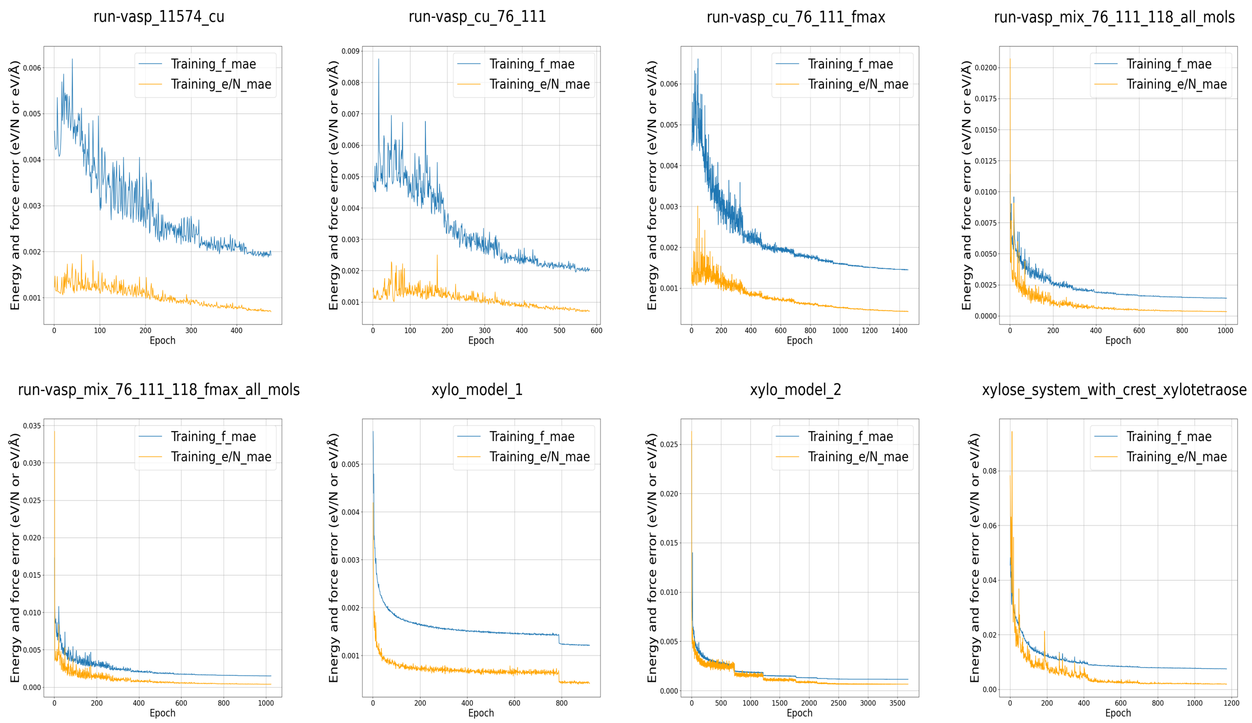

Figure S1: NequIP training force and energy mean average errors for the 8 potentials described in the main manuscript

All of the trained potentials reached relatively low force and energy mean average errors in as little as 400-500 training epochs (Figure S1). With the exception of "run-vasp_11574_cu" (1), and "run-vasp_cu_76_111" (2), the training progress had in principle converged to some value by the final training epoch.

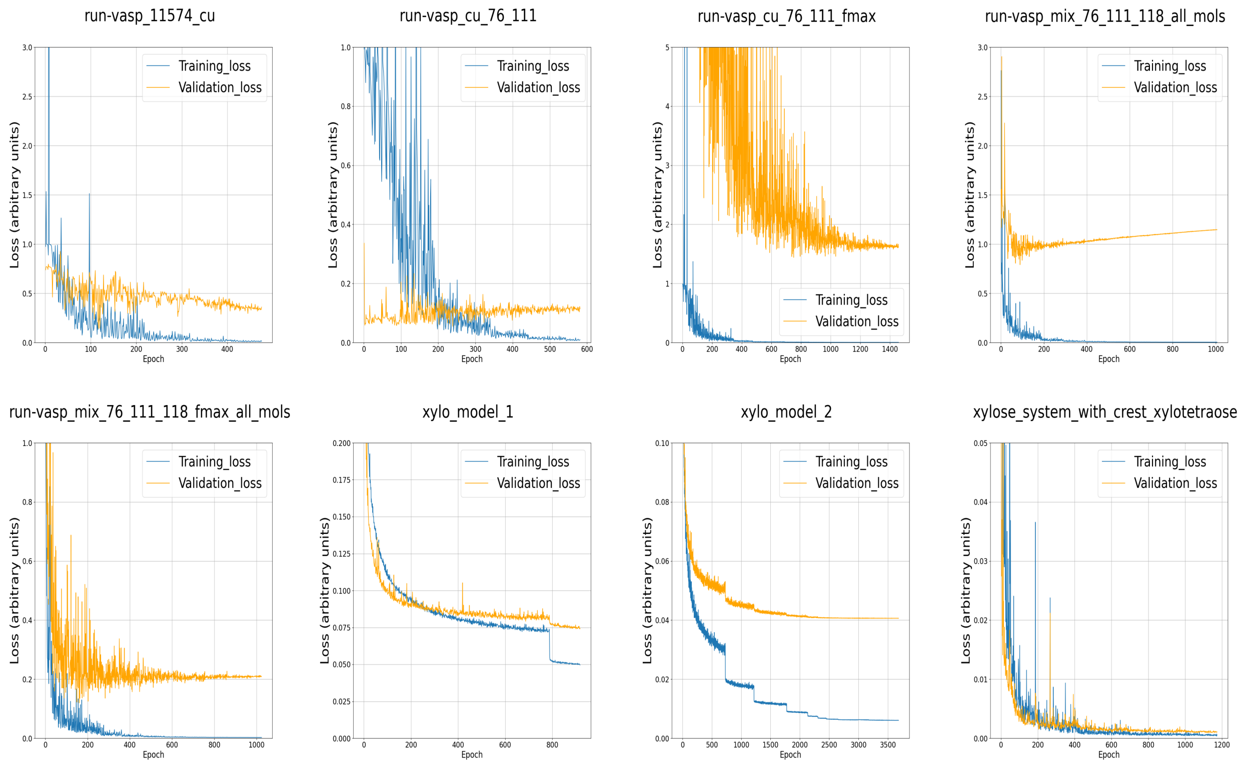

Figure S2: NequIP validation and training losses for the 8 potentials described in the main manuscript. Note the different scales on the y-axes

From inspection of the validation and training losses, we note that potential 4, or ”run-vasp_mix_76_111_118_all_mols” shows clear signs of overfitting by the diverging loss curves.[S1,S2] It should be noted that the high validation loss for potential three is likely due to the validation set containing only high-energy adsorption structures, which the model has not seen during training.

1.2 Validation tests

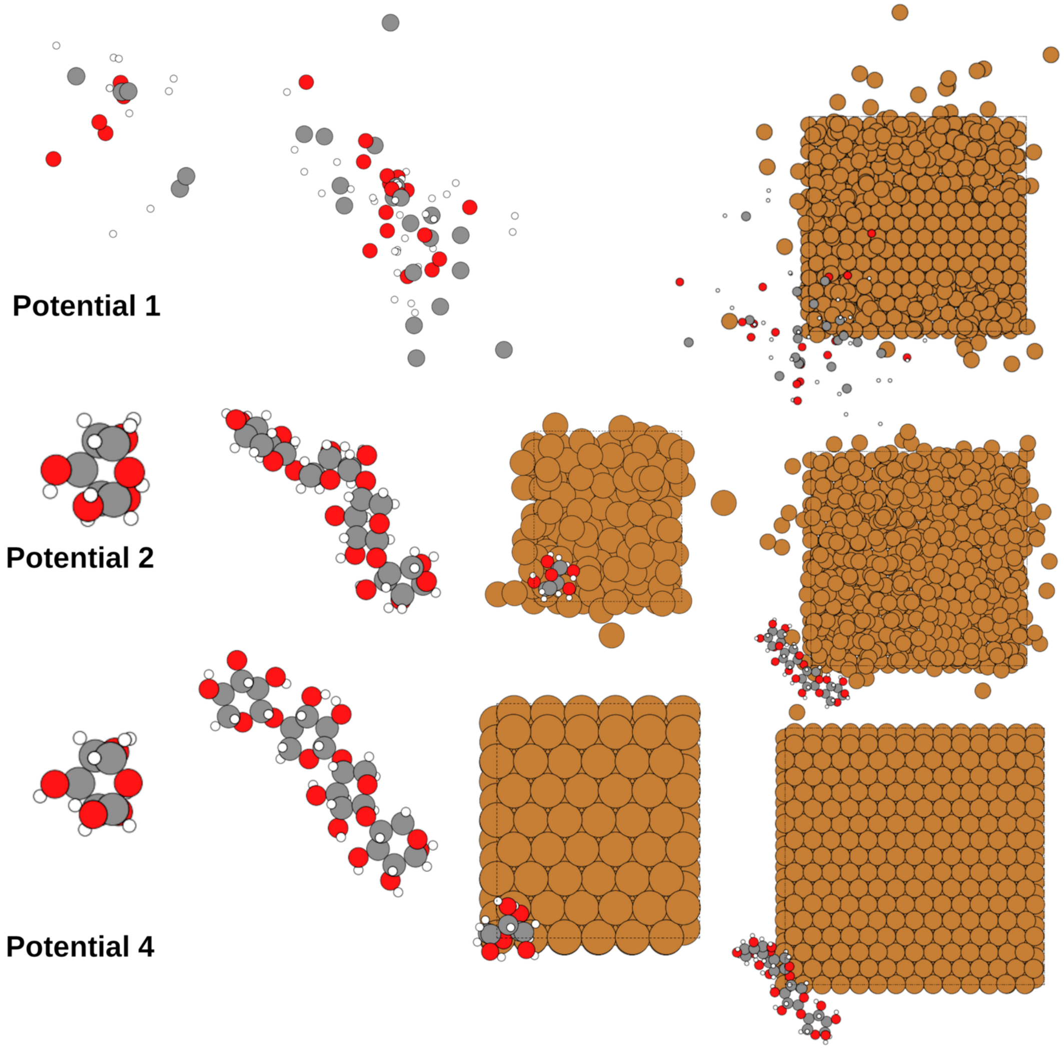

Figure S3: NequIP validation test relaxations 1

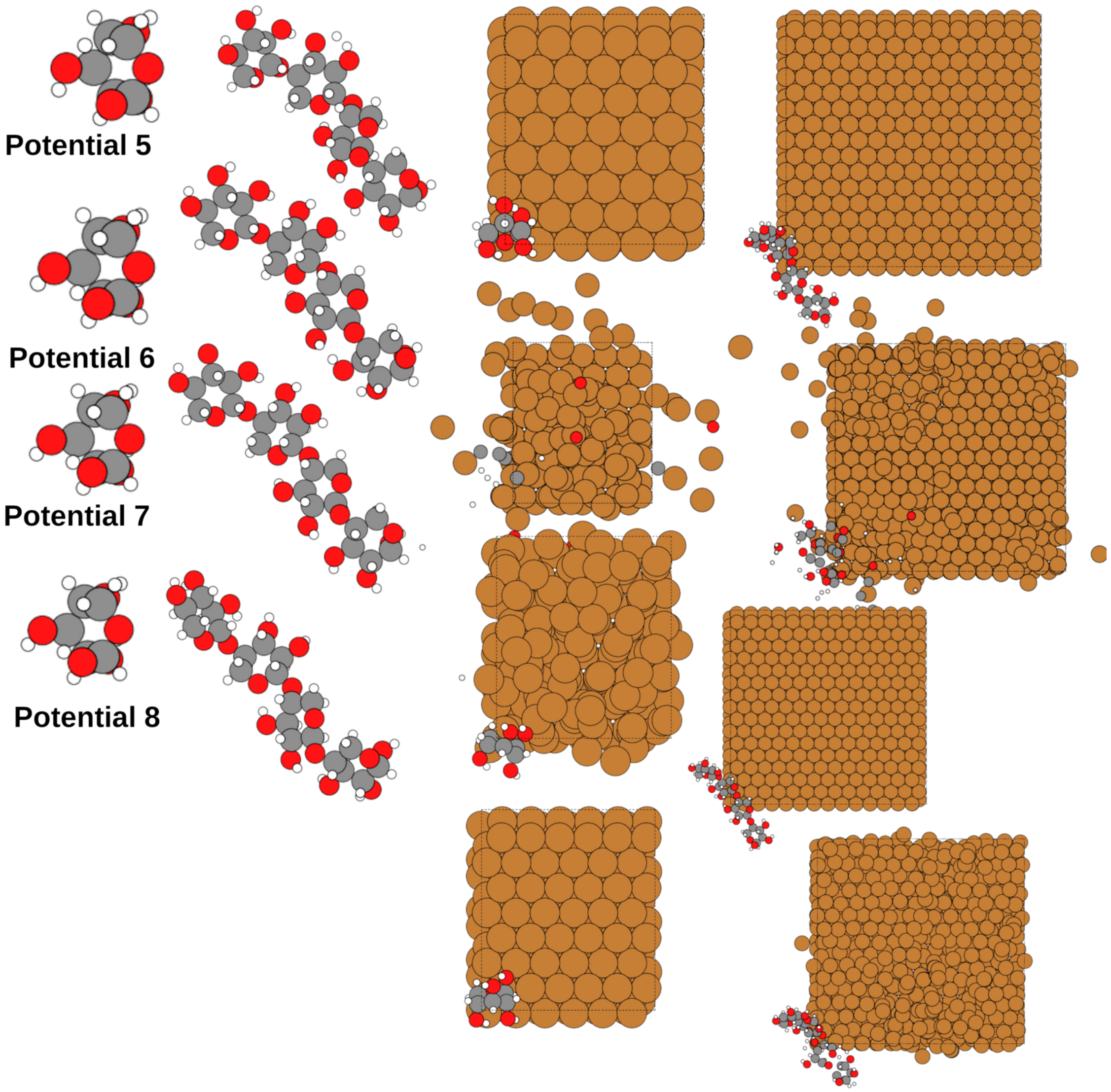

Figure S4: NequIP validation test relaxations 2

The validation tests already shown in the main manuscript are here shown for the remaining potentials 1, 2, and 4-8 in Figures S3 and S4. All of them except potential 1 are able to relax isolated molecules, although some variations are observed in the final geometries for each one. As stated in the main manuscript, the issues experienced with the surface relaxation could usually be remedied by constraining the surface during the process.

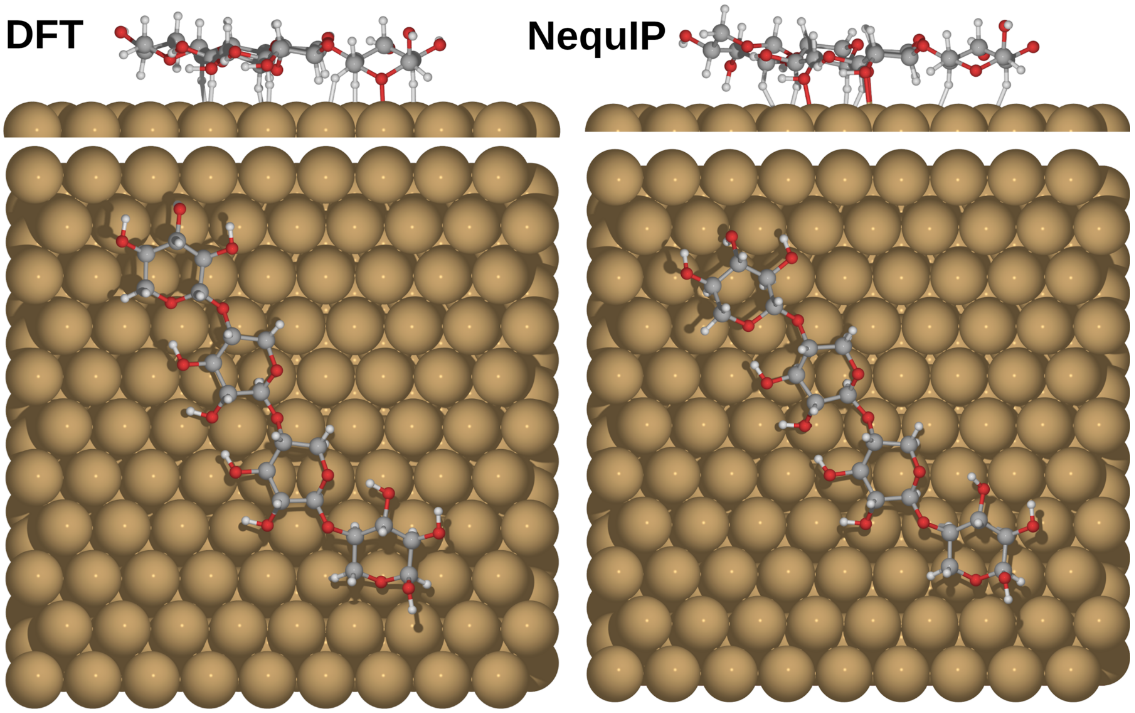

Figure S5: DFT and NequIP potential 8 relaxation comparison. This is the same xylotetraose geometry as shown in the main manuscript under validation tests

As seen in Figure S5, potential eight manages to relax the xylotetraose adsorbate to the surface in the same manner as DFT. Although the two structures still differ, certain features are maintained, for instance the hydrogen-bonding network. Potential 8 also differs from potential 3 in their distance cutoff values, being 3.5 and 4.5 Å. This indicates that inclusion of interactions between slightly more distant groups could also be a relevant factor as to whether the whole chain relaxes to the surface or not.

2 BOSS

2.1 Xylose conformer analysis surrogate model

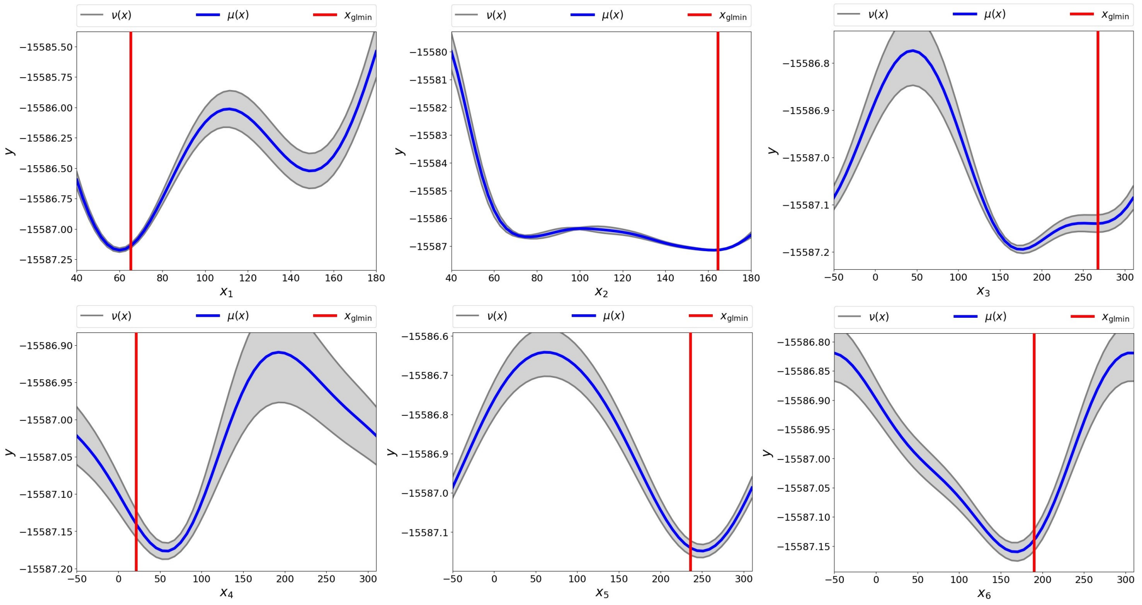

Figure S6: BOSS surrogate model for the xylose conformational PES. X_1 = ring O_1-puckering (X_1-X_2-O_1 angle), X_2 = C_3-puckering (X_1-X_3-C_3 angle), X_3 = OH_2 rotation (H_2-O_2-C_1-C_2 dihedral angle), X_4 = OH_3 rotation (H_3-O_3-C_2-C_1 dihedral angle), X_5 = OH_4 rotation (H_4-O_4-C_3-X_3 dihedral angle), X_6 = OH_5 rotation (H_5-O_5-C_4-C_5 dihedral angle)

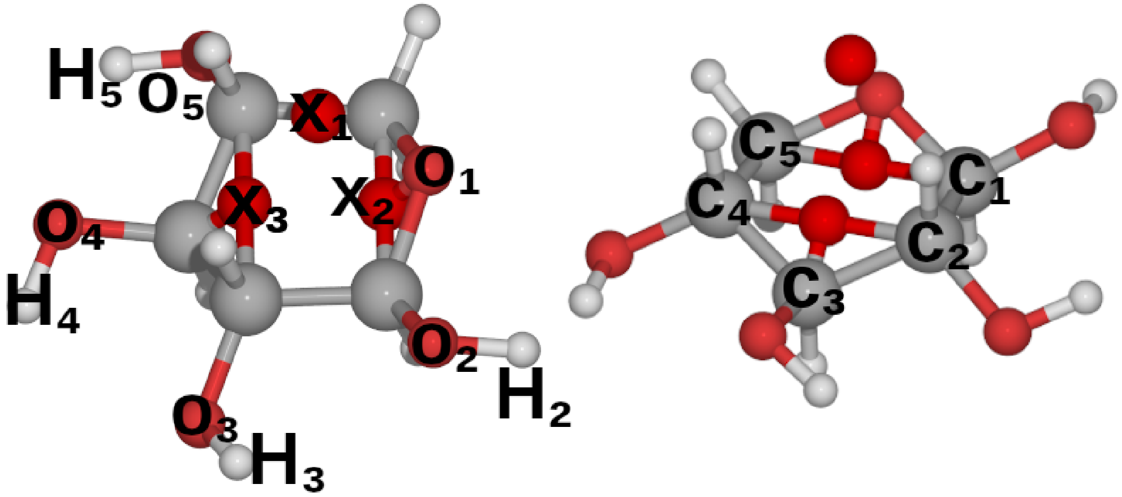

Figure S7: xylose atomic labels

As displayed in Figure S6, the predicted BOSS global minimum variables are as follows: $X_1 = 65°$, $X_2 = 165$ °, $X_3 = 267°$, $X_4 = 21°$, $X_5 = 236°$ and $X_6 = 190°$, corresponding to the BOSS local minimum structure 1 ($^4C_1$-chair) in the main manuscript. The relevant atomic labels are shown in Figure S7. From the 1D potential energy surface of X_2, we note how higher energies for some of the variables in the search lead to slightly heightened uncertainties in the rest. This is the reason we employed the energy transformation method described by Fang and coworkers[S3] to augment the high-energy regions during our subsequent structure searches. However, we did not find this necessary for this particular system, as we validated these structures with literature.[S4] From the surrogate models, the estimated barrier for changing the ring-configuration from the most stable $^4C_1$-chair to a boat conformer is about 1.2 eV, while the OH-rotation barriers are 0.5 eV at most. However, it should be noted that if one of the variables change, all of the 1D profiles corresponding to the other variables change with it, and thus the latter are only crude estimates.

2.2 Xylose conformer analysis with $^1C_4$-initial structure

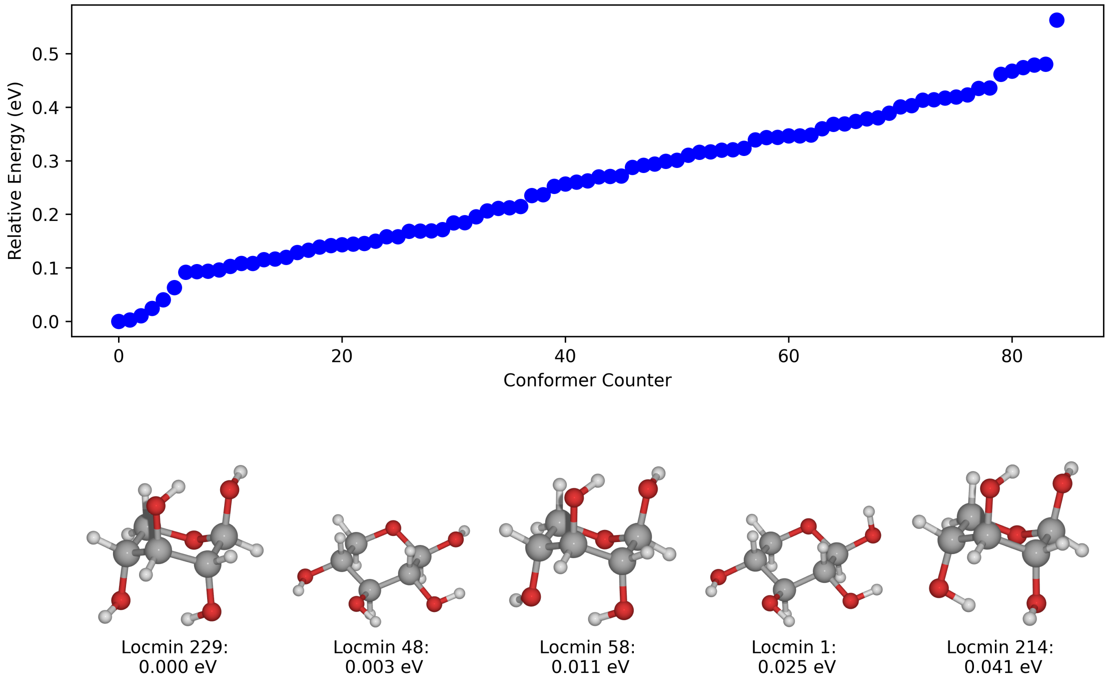

Figure S8: Relative energies of the PBE+vdW^{surf} relaxed BOSS (414 data points were used to construct the surrogate model) predicted conformers of β-D-xylose. The five most stable conformers from each method are displayed in order of increasing energy from left to right. The energy ordering of the predictions equals the shown conformer indices

Figure S8 shows the results from a BOSS conformer analysis using a different initial structure ($^1C_4$) than described in the main manuscript. Although the ordering of the predicted conformers change slightly, the overall trends are maintained in terms of the five most stable structures. In fact, using the $^1C_4$-structure as the starting point of the structure search results in an identical distribution of the five most stable conformers as the CREST-based search.

3 CREST

3.1 α- and β-terminated xylotetraose

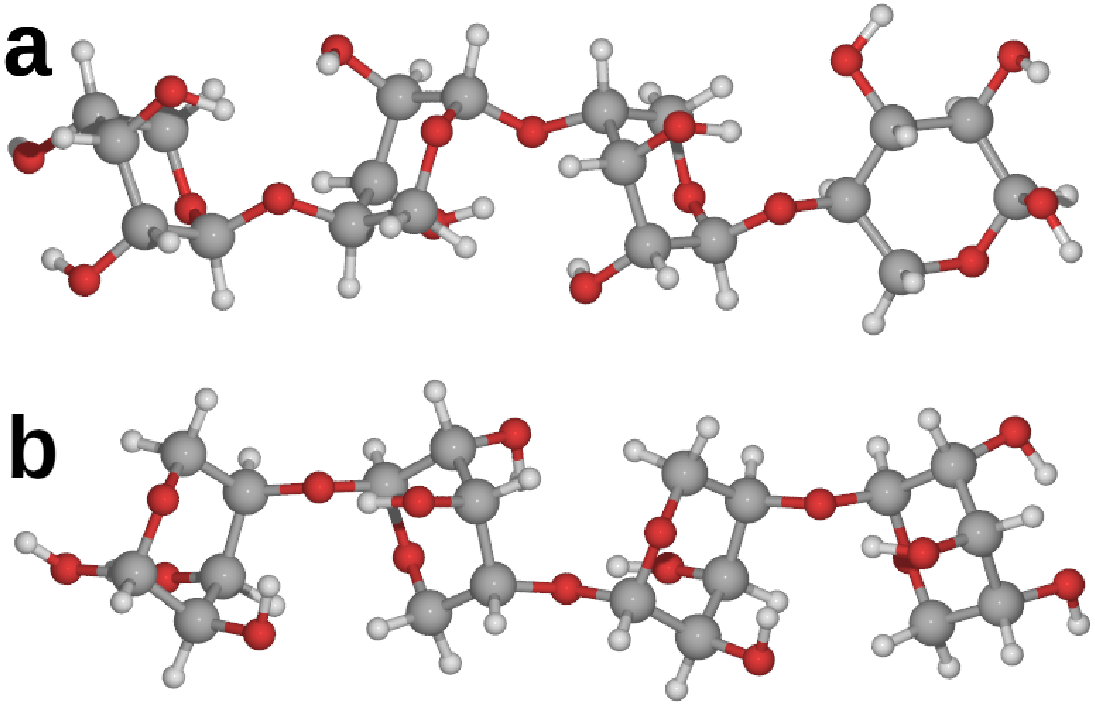

Figure S9: CREST predicted lowest energy xylotetraose anomers with a) α- and b) β-termination

The most stable configurations of xylotetraose differing in anomeric termination are displayed in Figure S9. According to our CREST analysis (no DFT relaxation), the most stable α-terminated xylotetraose has one xylose unit is a $^4C_1$-chair, the rest being $^1C_4$. At the same time, the whole chain of the β-terminated counterpart is made up of $^1C_4$-chair units. When relaxing the CREST conformers with DFT, the global minima change as illustrated in Figure S10. The α-conformer has two xylose units in $^1C_4$, two in $^4C_1$ configurations. In contrast, the β-conformer has three units in $^1C_4$, one in $^2S_0$. This is illustrative of how the optimal H-bonding network changes with a single positional substitution. Another distinction between xylotetraose with different chair forms is where the OH-bonds prefer to orient. With $^4C_1$,

the OH-bonds are oriented around the individual xylose units, while for the ${}^1C_4$ species, these are mainly oriented towards the opposite glycosidic bond oxygen atoms.

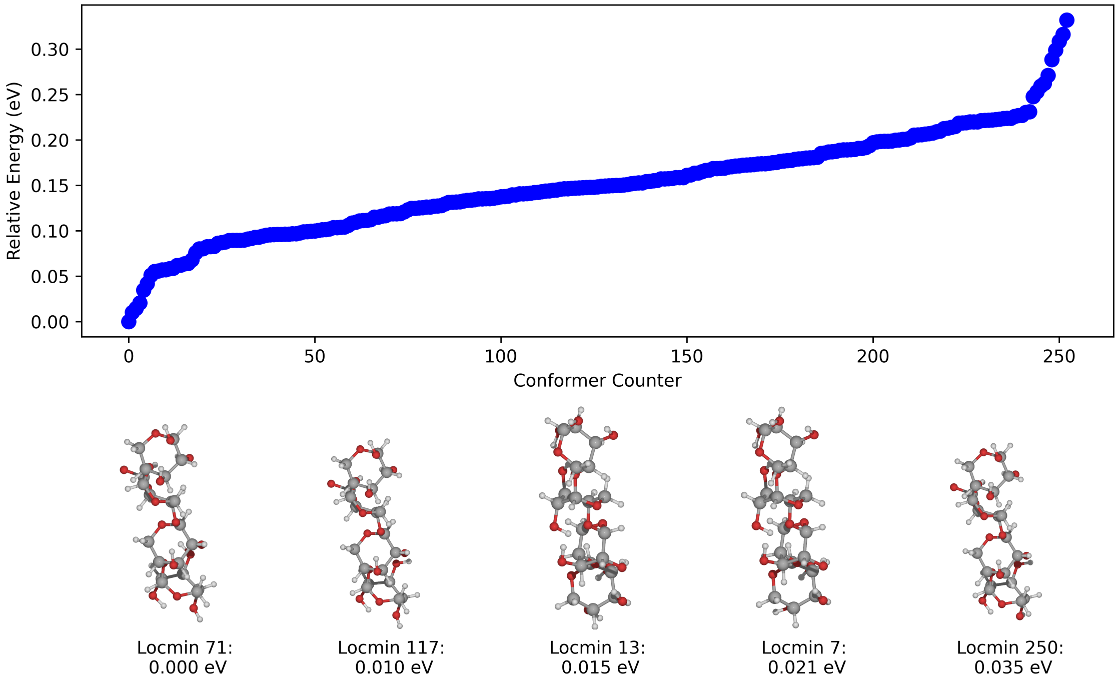

Figure S10: Relative energies of the PBE+vdW^{surf} relaxed CREST predicted conformers of β-terminated 1,4-β-D-xylotetraose. The five most stable conformers from each method are displayed in order of increasing energy from left to right. The energy ordering of the predictions equals the shown conformer indices

4 DFT relaxations of BOSS (NequIP) global minima

4.1 Xylose on Cu111

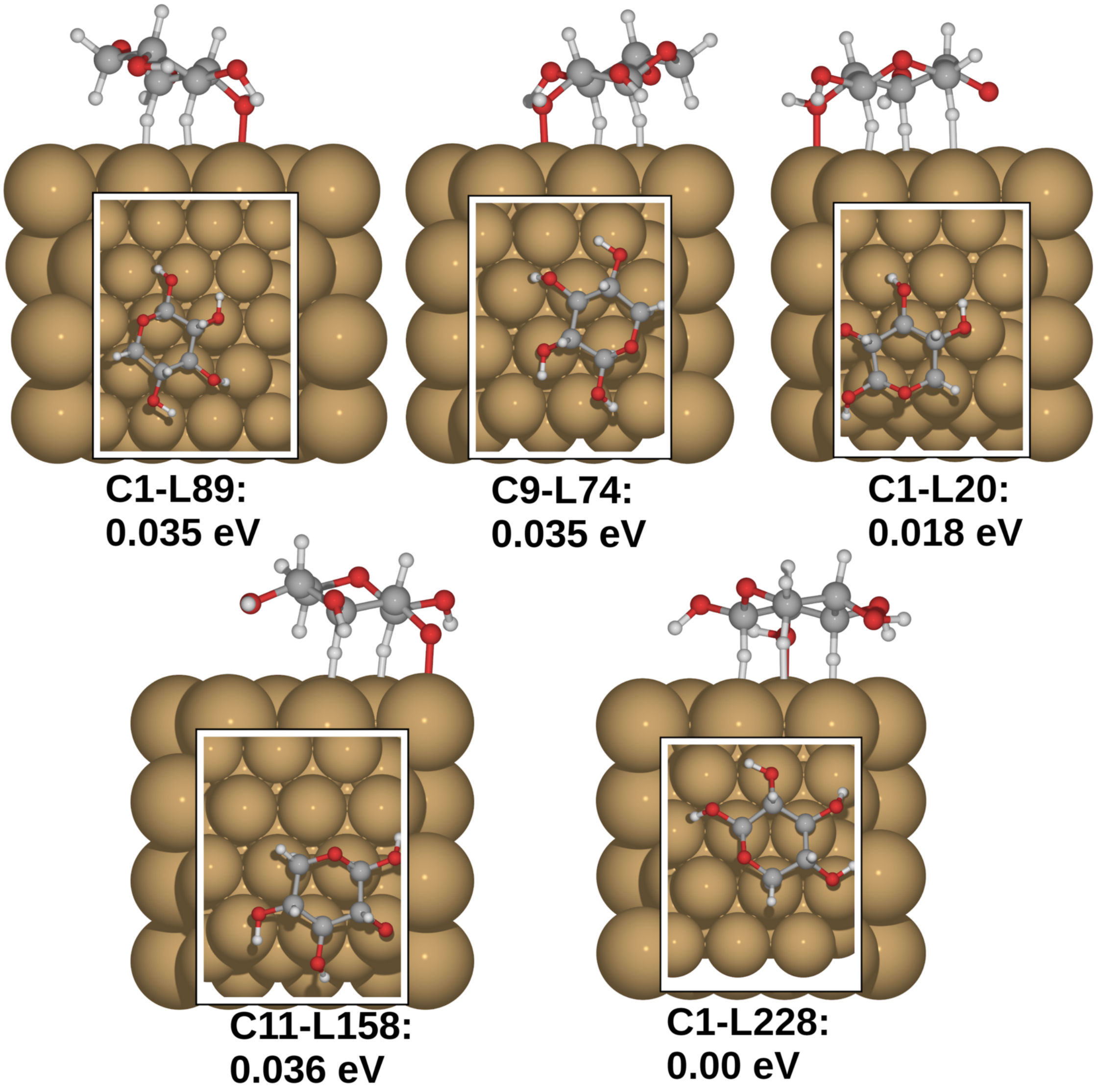

Figure S11: DFT relaxations and corresponding relative energies of the BOSS (NequIP) xylose adsorption global minima.

DFT (PBE+vdW^{surf}) relaxation of the BOSS (NequIP) lowest energy structures are shown in Figure S11. While most of the energies are close in agreement with the NequIP energy order, a couple of the structures deviate from the trend. For instance, the C1-L20 structure

is slightly lower in DFT energy while NequIP places it higher. Furthermore, the C1-L228 structure relaxes to a structure that is even lower than what the DFT-based BOSS search provided, hence representing the global adsorption minimum for xylose on copper. Although it resembles the predicted global minimum, C1-L89, the axial endocyclic hydrogen atoms are closer to the top position of the surface Cu, and the adsorbate is aligned with the surface in contrast to being tilted towards one of the OH-groups as the former.

4.2 Xylotetraose on Cu111

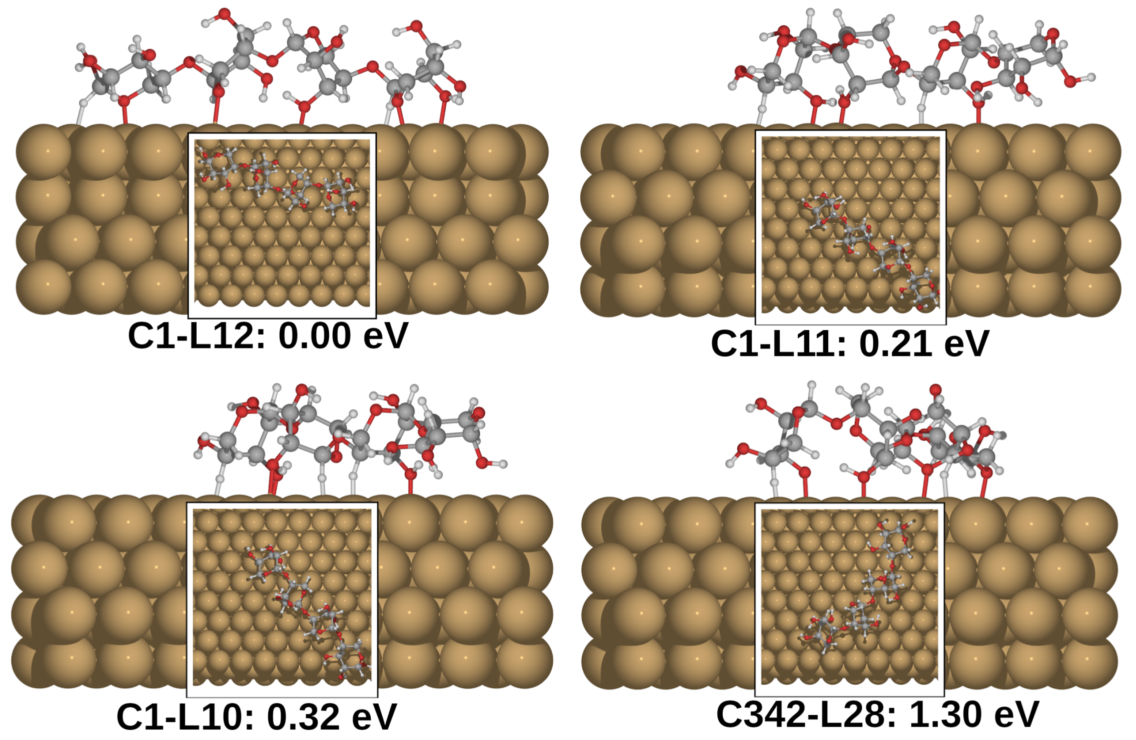

Figure S12: DFT relaxations and corresponding relative energies of the BOSS (NequIP) xylotetraose adsorption global minima.

DFT (PBE+vdW^{surf}) relaxation of the BOSS (NequIP) lowest energy structures are shown in Figure S12. The relative energy order of the structures shown here is in agreement with NequIP, although the absolute values (0.00, 0.03, 0.07 and 0.13, respectively) deviate. During DFT relaxation, the most drastic change in the structures is generally the elongation of the

xylotetraose moiety, while the adsorption height and relative positioning of the adsorbate on the surface are fairly well maintained.

References

(S1) Srivastava, N.; Hinton, G.; Krizhevsky, A.; Sutskever, I.; Salakhutdinov, R. Dropout: A Simple Way to Prevent Neural Networks from Overfitting. *Journal of Machine Learning Research* **2014**, *15*, 1929–1958.

(S2) Ying, X. An Overview of Overfitting and its Solutions. *Journal of Physics: Conference Series* **2019**, *1168*, 022022.

(S3) Fang, L.; Makkonen, E.; Todorović, M.; Rinke, P.; Chen, X. Efficient Amino Acid Conformer Search with Bayesian Optimization. *Journal of Chemical Theory and Computation* **2021**, *17*, 1955–1966, Publisher: American Chemical Society.

(S4) Peña, I.; Mata, S.; Martín, A.; Cabezas, C.; M. Daly, A.; L. Alonso, J. Conformations of d -xylose: the pivotal role of the intramolecular hydrogen-bonding. *Physical Chemistry Chemical Physics* **2013**, *15*, 18243–18248, Publisher: Royal Society of Chemistry.
